# Polymer-Supported Phosphoric, Phosphonic and Phosphinic Acids—From Synthesis to Properties and Applications in Separation Processes

**DOI:** 10.3390/molecules25184236

**Published:** 2020-09-15

**Authors:** Agnieszka Głowińska, Andrzej W. Trochimczuk

**Affiliations:** Department of Polymer Engineering and Technology, Wroclaw University of Science and Technology, Wybrzeże Wyspiańskiego 27, 50-370 Wrocław, Poland; agnieszka.glowinska@pwr.edu.pl

**Keywords:** organophosphorus polymers, phosphorus-containing polymers, ion exchange resins, chelating resins, separation processes

## Abstract

Efficient separation technologies are crucial to the environment and world economy. The challenge posed to scientists is how to engineer selectivity towards a targeted substrate, especially from multicomponent solutions. Polymer-supported reagents have gained a lot of attention in this context, as they eliminate a lot of inconveniences concerning widely used solvent extraction techniques. Nevertheless, the choice of an appropriate ligand for immobilization may be derived from the behavior of soluble compounds under solvent extraction conditions. Organophosphorus compounds play a significant role in separation science and technology. The features they possess, such as variable oxidation states, multivalence, asymmetry and metal-binding properties, highlight their status as a unique and versatile class of compounds, capable of selective separations proceeding through different mechanisms. This review provides a detailed survey of polymers containing phosphoric, phosphonic and phosphinic acid functionalities in the side chain and covers main advances in the preparation and application of these materials in separation science, including the most relevant synthesis routes (Arbuzov, Perkow, Mannich, Kabachnik-Fields reactions, etc.), as well as the main stages in the development of organophosphorus resins and the most important achievements in the field.

## 1. Introduction

Beside the ubiquitous role they serve in nature, phosphorus-containing compounds have found use in numerous fields, especially chemistry and biochemistry [1]. The polymer chemistry of phosphorus is based mostly on compounds possessing stable C-P bonds or ones that are inorganic acids—e.g., phosphonic (R-P(O)(OH)_2_), phosphoric (R-O-P(O)(OH)_2_) and phosphinic (R-P(H)(O)(OH)) acid or their derivatives [2]. In comparison to other organic polymers, the number of those phosphorus-containing species is relatively low. The first known synthetic one was polyphosphazene, known also as an inorganic rubber, reported by Stokes in 1897. At the beginning, its technological potential was not fully appreciated [3]. However, in the last few decades we could observe an increasing attention to organophosphorus polymers, which is undoubtedly associated with their important role in science and technology.

The main reason for the uniqueness of such polymers lies in their diversified properties, which can be partially explained, i.a., by the ionization potential of phosphorus-containing acids [4,5]. Numerous comprehensive works—review articles and books regarding organophosphorus polymers were published over the years. Undoubtedly, one worth-mentioning is *Phosphorus-Based Polymers—From Synthesis to Applications* edited by Monge and David [5], as it covers the development and application of phosphorus-containing polymers in diversified areas—i.a., the biomedical field, anticorrosion compounds, flame retardancy, removal of metal ions from wastewater, etc.

As described in many reports, the presence of phosphorus in the polymer chain is advantageous—it imparts better flame retardancy, improves dyability, provides higher antistatic properties, enhances solubility, adhesion, etc. [3]. Moreover, it has been established that phosphorus-bearing compounds are able to increase the solubility (hydrophilicity), adhesion, biocompatibility and biodegradability of materials [5].

Among many possible applications, phosphorus-containing resins employed in separation processes deserve particular attention. Several important stages may be distinguished considering the progress in this area—e.g., the expansion of experiments concerning incorporation of phosphorus-containing functional groups, the requirement for actinides and lanthanides (nuclear energy, production of electronic devices, etc.), the development of bifunctional resins (one phosphorous containing groups and an additional one causing synergistic effects, adjusting the resin’s hydrophilicity or the distance between the functional groups, etc.).

This article provides an overview of polymers containing phosphoric, phosphonic and phosphinic functional groups in the side chain, collecting the most relevant synthesis routes and properties which enable these three groups of materials to be applied in separation processes. The pioneer works in the field have been highlighted and shortly discussed. Moreover, brief historical background—the main stages in the development of the organophosphorus resins have been presented.

## 2. Uniqueness of Phosphorus-Containing Compounds

Phosphorus may form many types of compounds: trivalent (lone pair present, pyramidal), tetravalent (hybridization sp^3^, tetrahedral), pentavalent (hybridization sp^3^d, trigonal bipyramidal) and hexavalent (hybridization sp^3^d^2^, octahedral). The 3s→3d promotion energy is low enough for the vacant d-orbitals to participate in chemical bonding, whereas in the case of nitrogen, the 2s→3d promotion energy is too large for effective d bonding. The high energy d-orbitals contribute to reduced electronegativity and greater polarizability of phosphorus when compared to other elements, such as N. Phosphorus compounds react mostly by a pair mechanism—due to the nucleophilic reactivity of the lone pair electrons (trivalent compounds) or phosphorus electrophilicity (pentavalent derivatives) [3].

The atomic orbitals of phosphorus atoms overlap with those having sufficient electron density. Different types of bonding may be formed—σ-bonding as well as multiple π-bonding, e.g., p_π_(P)-p_π_(X), -d_π_(P)-p_π_(X), -d_π_(P)-d_π_(X) where X stands commonly for carbon, nitrogen or oxygen. However, polymers containing P-O-C, P-O-P, P-N linkages are rather hydrolytically unstable, contrary to those possessing P-C linkages [3]. The stability of a given bond is guaranteed by the bond strength and is dependent on the atmosphere to which it is subjected. High bond strength does not guarantee high stability of the compounds.

For example, based on a given values of bond energies (Table 1), polymers possessing P-C, P-O and P-N linkages in main chain are expected to be thermostable. It is also worth to notice that P-O bond is stronger that N-O or C-O bonds. The P-O, P-C or P-N linkages are generally stronger than in the cases where phosphorus is replaced with nitrogen. Moreover, P-S linkages reduce hydrolytic and thermal stability of the compound when compared to P-O [3].

Among many possible applications, phosphorus-containing resins employed in separation processes deserve exceptional attention. The main reason for their uniqueness lies in their diversified properties, which can be partially explained by the ionization potential of phosphorus-containing acids, which lies between that of sulfonic and carboxylic acids as a result of their intermediate pKa values [4,5,6]. Phosphoric acid (H_3_PO_4_) has three ionization equilibria, as follows: H_3_PO_4_ → H^+^ + H_2_PO_4_^−^ (pKa_1_ = 2.16), H_2_PO_4_^−^ → H^+^ + HPO_4_^2−^ (pKa_2_ = 7.21) and HPO_4_^2−^ → H^+^ + PO_4_^3−^ (pKa_3_ = 12.32), phosphonic acid has two (pKa_1_ = 1.3, pKa_2_ = 6.70) and phosphinic acid one (pKa = 1.2), whereas e.g., sulphonic acid is dissociated in whole pH range, acetic acid is dissociated above pKa = 4.7. This feature provides unique properties of phosphorus compounds in separation processes—above their pKa, they are able to bind metals through ion-exchange, below it —through a complexation mechanism. Thus, they are more selective than the strongly acidic polymers and can be used over a wider pH range [7]. They are proved to be highly selective toward so-called hard Lewis acid cations in the hard and soft acid and base (HSAB) theory, especially lanthanides, U(VI), Ti(IV) and Fe(III) [6].

Moreover, phosphorus-based ligands are well suited for reactive ion exchange [7]. This term was first coined by Janauer [8], who explained that chemical reactions such as oxidation, reduction, formation of sparingly soluble compounds, neutralization, ion complexing and chelation reactions may be employed to “drive” ion exchange processes in the desired direction.

Furthermore, one of the most important features of organophosphorus compounds is the presence of the phosphoryl group in their structure. This group exhibits many advantages, among which of great importance remain its ease of formation, thermal stability and resistance to chemical modification. The stability of P=O group is derived from a form of multiple bonding, which can be improved in the presence of strongly electronegative groups on phosphorus. The phosphoryl group is present in a range of very stable acidic structures (e.g., described herein as phosphonic (R-P(O)(OH)_2_), phosphoric (R-O-P(O)(OH)_2_) and phosphinic (R-P(H)(O)(OH)) acids). As has been proven in many literature reports, structures with such functionalities play a significant role in organophosphorus chemistry. This topic has been widely studied and described in literature [2,5].

## 3. Resins Preparation

**Scheme 1:**
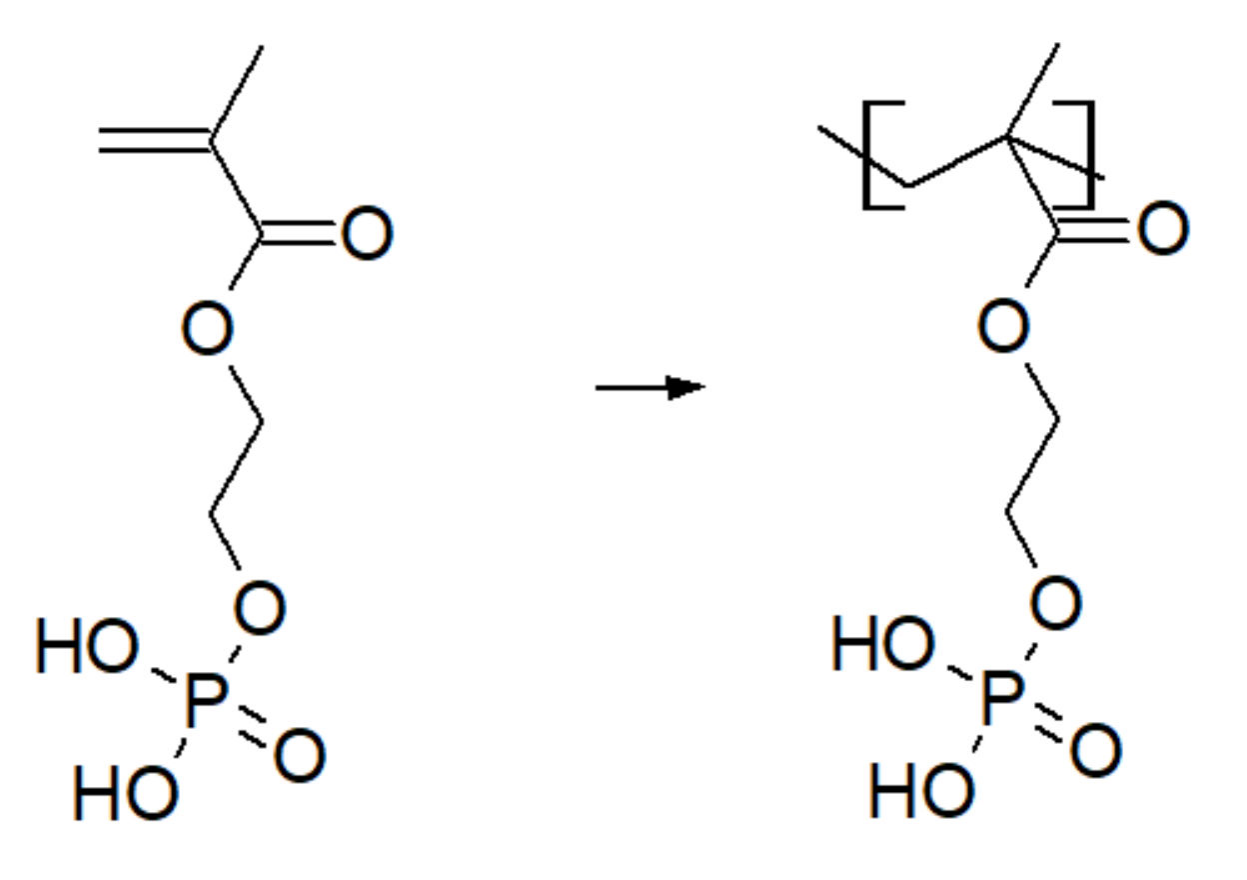
Synthesis of a phosphoric acid polymer by direct polymerization of functionalized monomer based on the example of 2-methacryoyloxyethyl phosphate.

Organophosphorus polymers may be divided into three groups: polymers possessing phosphorus: (i) only in the main chain, (ii) in side chains only, (iii) cyclic structures within the main chain [3]. There are several strategies to introduce phosphorous-containing moieties into the polymeric backbone and they consist of direct polymerization of monomers with such groups (Scheme 1) or chemical modification of allyl and vinyl monomers [9].

**Scheme 2:**
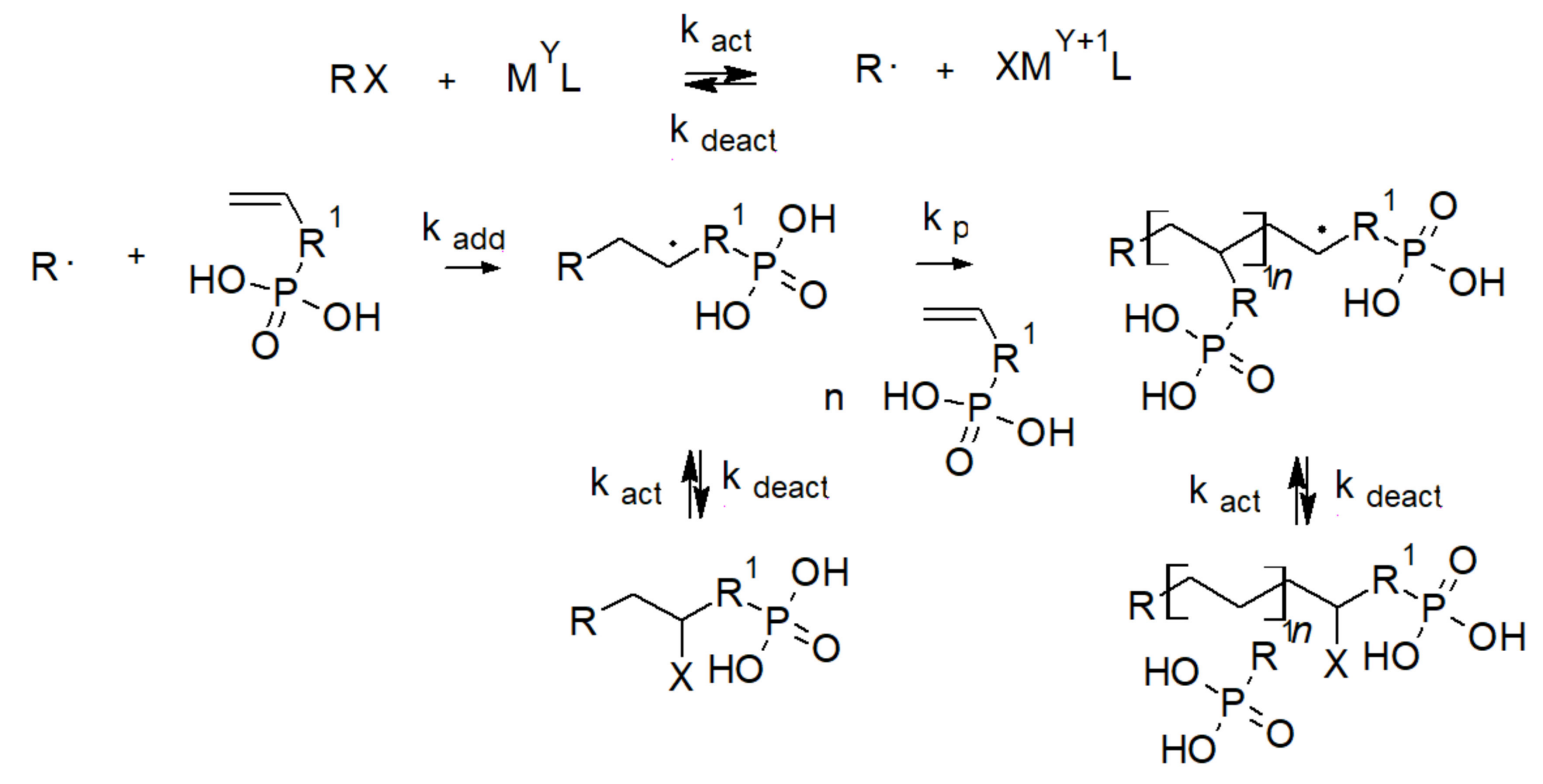
Schematic representation of Atom Transfer Radical Polymerization (ATRP) (X—halide: Br or Cl, M—transition metal, L—complexing ligand, Y—oxidation state, k_act_—activation rate constant, k_deact_—deactivation rate constant, k_p_ —propagation rate constant) based on the example of phosphonic acid monomer.

**Scheme 3:**
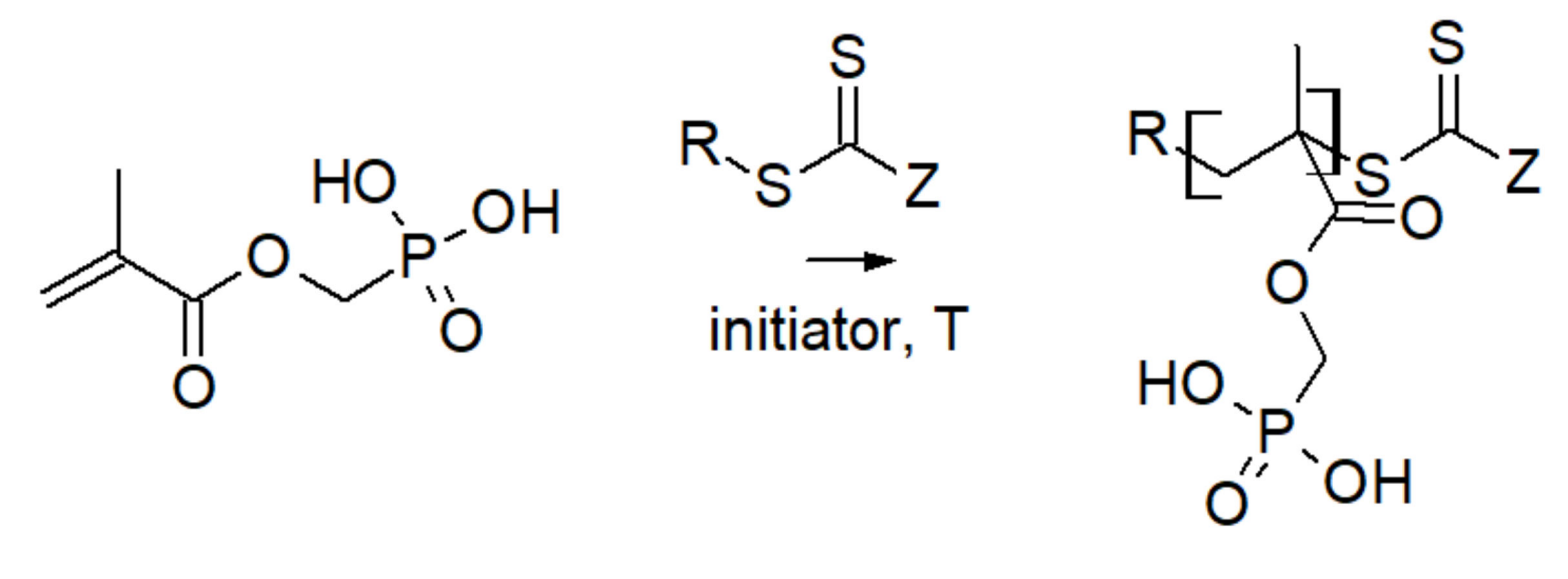
Schematic representation of a reversible addition fragmentation transfer (RAFT) process based on the example of methacryloyloxymethyl phosphonate using dithioester chain transfer agent (Z—a group that modifies addition and fragmentation rates, R—a good homolytic leaving group and a good initiating species).

Concerning synthesis methods, which are common for groups described herein, free radical polymerization and photopolymerization have been the most studied, but there are also reports of controlled radical polymerization: atom transfer radical polymerization (ATRP) [10,11,12,13,14,15,16,17,18] (Scheme 2) or reversible addition fragmentation transfer (RAFT) processes [6,19,20,21,22,23,24] (Scheme 3). Living polymerization techniques allow one to obtain well-defined polymers with tunable structures, compositions, and properties [5]. What is interesting, phosphorus-bearing compounds can also play an important role as intermediates (chain transfer agents) for RAFT polymerization. Uchiyama et al. [25] used phosphoric and phosphinic acid derivatives in the synthesis of polymers with controlled molecular weights and obtained materials with M_n_ up to ~1 × 10^5^ and narrow molecular weight distributions (M_w_/M_n_∼1.1).

**Scheme 4:**
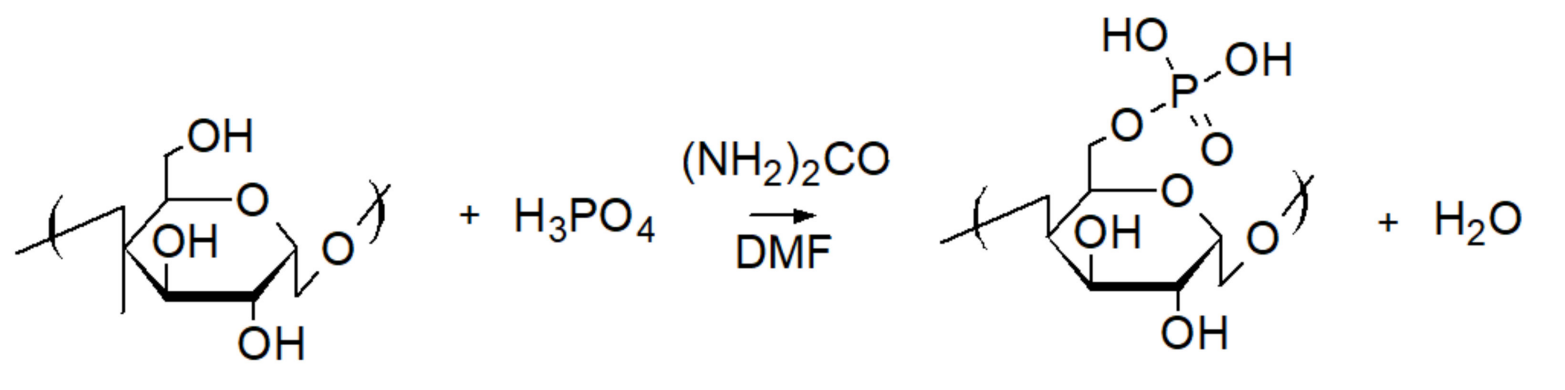
Schematic representation of cellulose phosphorylation [5].

Polymer-supported reagents can be successfully applied in metal separation, however it requires a fundamental understanding of metal-ligand interactions, the role of polymer in the whole process and the importance of the synthesis method [26]. The effectiveness of the polymer-supported resins, regardless of the type of ligand, strongly depends on parameters such as matrix rigidity determined by the crosslinking degree as well as the type of crosslinking agent, morphology and particle size [4,27]. Matrix porosity is also a factor of great importance, as it determines accessibility into the polymer matrix and capacity of the ligand [4]. Jyo et al. further proved the importance of crosslinking agent on resin selectivity towards different metal ions [28]. It is worth emphasizing here that synthetic polymers are not the only possibility. Modified natural polymers are often chosen as a matrix, mostly for ecological reasons. Adsorption of metal ions on cellulose phosphate has been studied for decades [29,30,31,32,33,34]. Phosphorylated cellulose was successfully applied in the rapid removal process of divalent and trivalent ions, such as Cu^2+^, Zn^2+^, Co^2+^, Ni^2+^, Pb^2+^, Mn^2+^, Fe^2+^, Fe^3+^, Cr^3+^ and lanthanides [5]. Modification of natural polymers was an extensively studied approach for the preparation of phosphorus-containing resins (e.g., cellulose, chitin and chitosan). An exemplary phosphorylation process is shown in the Scheme 4.

Although modified cotton fabrics exhibit ion exchange properties similar to those of the ion exchange resins, most of them were made primarily for other purposes, e.g., flame retardancy [32]. Cation exchange cottons can be prepared by the method of Ford and Hall [35] by curing cotton with a mixture of phosphoric acid and urea [32], but since 1949 various phosphorylating agents were studied for this purpose, i.e., mono- and dibasic ammonium phosphates, alkali metal phosphates and phosphonates [5], phosphorus oxychloride and sodium hydroxide [33] as well as pyridine [34]. Comparison between cation exchange capacities for different ion exchange resins and modified cotton fabrics have been provided elsewhere [32].

Chemical modification of chitin and chitosan with phosphonic acid or phosphonate groups influence their chelating properties and solubility, respectively [5]. Methods for their preparation have been described in the work of Jauakumar et al. [36] and include i.a., heating chitin or chitosan with orthophosphoric acid and urea in DMF, the reaction of chitin or chitosan with phosphorous pentoxide in methanesulphonic acid, modification by mixing chitin or chitosan with sodium pyrophosphate or by grafting mono(2-methacryloyl oxyethyl) phosphate onto chitosan. Depending on the type of modification, the obtained materials have potential to be applied not only as adsorbents, but also in drug delivery, biomedical field, food processing and tissue engineering [36].

In the following parts of this work (Section 3.1, Section 3.2 and Section 3.3) methods for exclusively obtaining polymer- supported phosphoric, phosphonic and phosphinic acids via modification of monomers or polymers resulting in introduction of the desired group into polymers will be described.

### 3.1. Phosphoric Acid Polymers

**Scheme 5:**
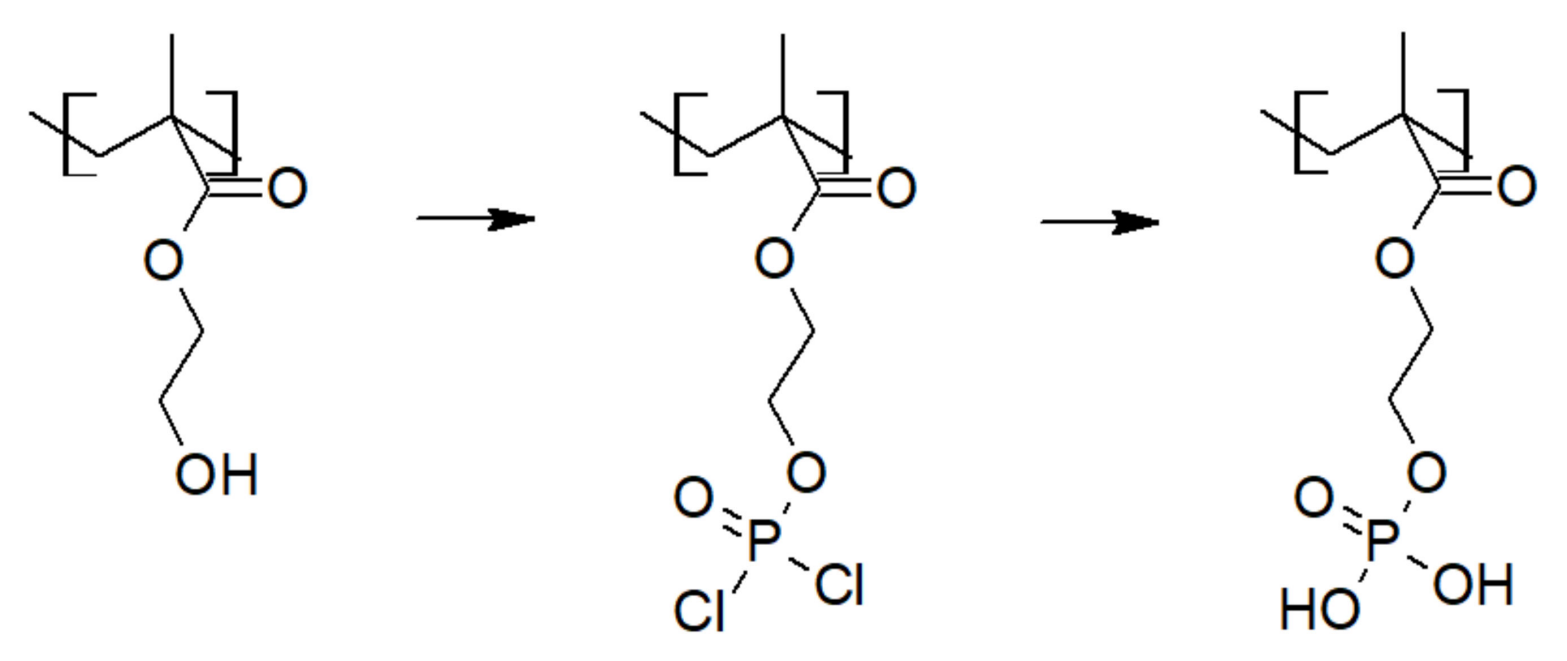
Phosphorylation with phosphoryl chloride based on the example of poly(2-hydroxyethyl methacrylate).

Polymer-supported phosphoric acids can be prepared by the phosphorylation of polymers (e.g., poly(vinyl alcohol), poly(2-hydroxyethyl methacrylate), poly(vinyl chloride)) with either phosphoric acid or phosphoryl chloride [4] (Scheme 5) or, as mentioned in the previous section of this work, by direct polymerization of the phosphoric acid monomer [4,37] (Scheme 1).

**Scheme 6:**
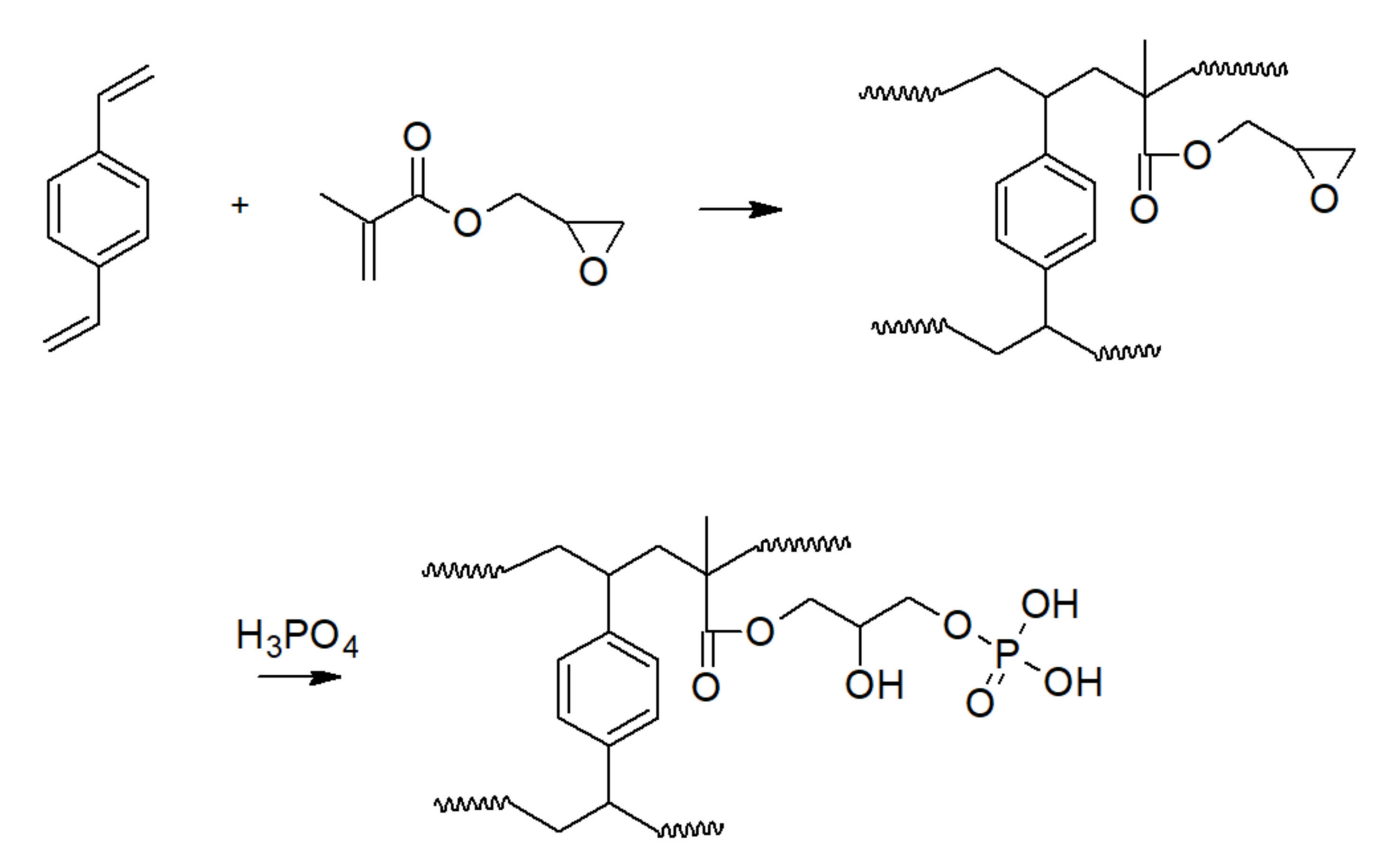
Preparation of phosphoric acid resin from poly(glycidyl methacrylate-co-divinylbenzene) [40].

Among the polymers mentioned earlier, crosslinked poly(glycidyl methacrylate) is of particular interest, since its functionalization can be easily performed by heating in phosphoric acid (Scheme 6). It was characterized and successfully applied for metal ions uptake [38,39,40]. Such resin can also be prepared using phosphoryl chloride, but this type of modification results in much worse cation exchange capacity, mainly because of the occurrence of inter- or intrapolymer bridging via phosphoryl acid groups during phosphorylation [40].

**Scheme 7:**
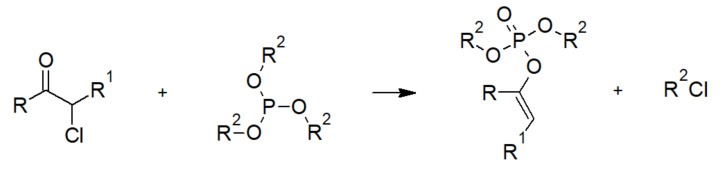
Schematic representation of Perkow reaction.

Phosphoric acid functionalities can also be attached to the polymer matrix via the Perkow reaction. In this type of synthesis, a trialkyl phosphite reacts with haloketone to form a dialkyl vinyl phosphate and an alkyl halide (Scheme 7). As the same reagents are able to form β-ketophosphonate in the Arbuzov reaction (described specifically in the next chapter) [41], the Perkow reaction is then considered as side-reaction.

**Scheme 8:**
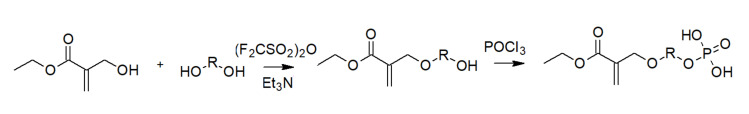
Schematic representation of a three-step synthesis resulting in monomers with phosphoric acid moieties [42].

Klee and Lehmann [42] synthesized novel monomers with phosphoric acid moieties via Baylis-Hillman reaction of ethyl acrylate and formaldehyde and subsequent etherification of the obtained product with diols followed by phosphorylation using POCl_3_ (Scheme 8). Methods for improving hydrolytic stability of monomers were described in literature, e.g., the presence of ether bond [6,43,44,45,46].

### 3.2. Phosphonic Acid Polymers

The most extensively studied group in recent years are phosphonate methacrylic acid derivatives, due to their high reactivity in radical copolymerization, contrary to allylic and vinylic derivatives, which, according to the literature [47,48], usually show low reactivity resulting in low molecular weight oligomers. It is also worth mentioning that only a limited number of phosphoric esters have been reported in literature, since their hydrolysis is easier in comparison with phosphonate derivatives [5].

There are many reports of introducing phosphonic acid moieties, but most of them consider two strategies: chemical modification of the monomers or the polymer side-chains. Organophosphorus polymers based on P-C linkages are generally more difficult to prepare than those with, e.g., P-O or P-N linkages [3].

**Scheme 9:**
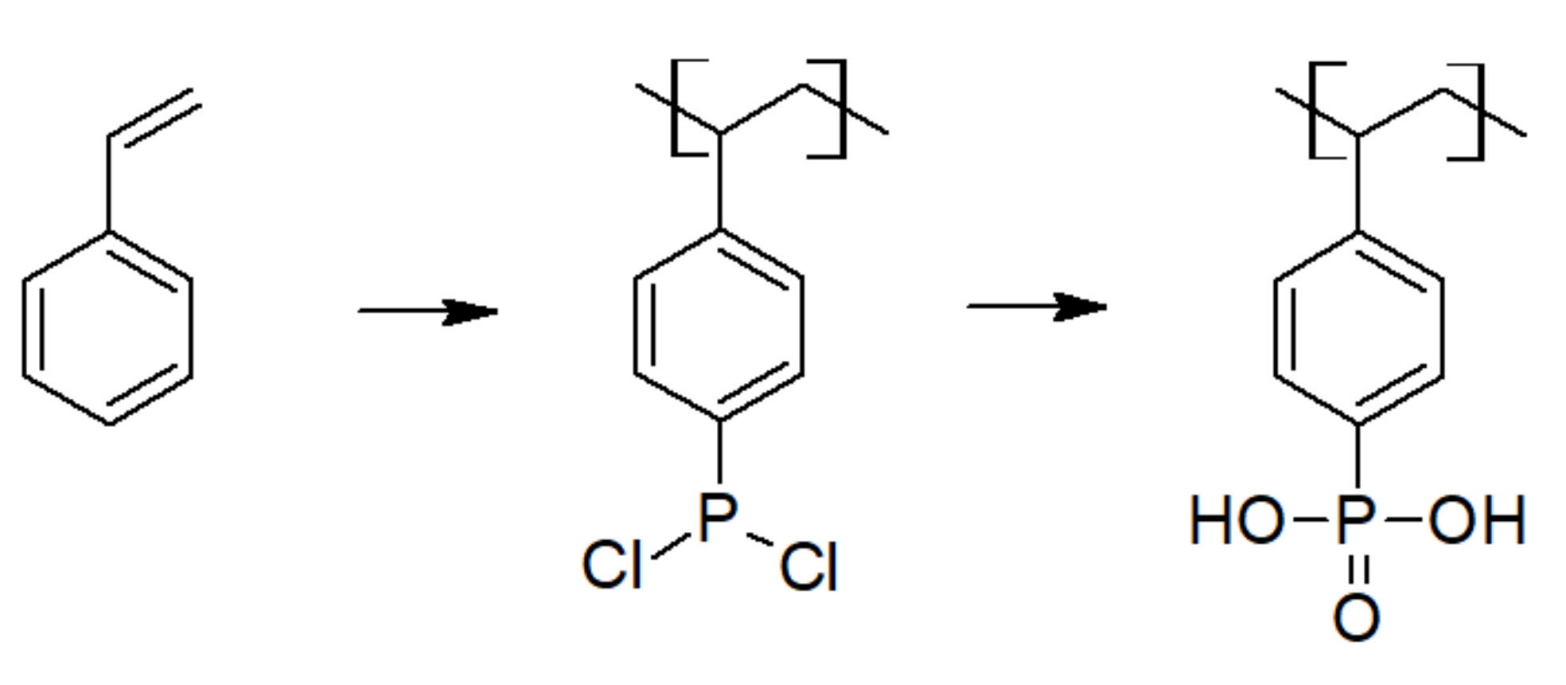
Synthesis of phosphonic acid polymers via Friedel-Crafts reaction based on the example of polystyrene-employing reaction.

Polymer-supported phosphonic acids can be prepared by the functionalization with PCl_3_/AlCl_3_ (Friedel-Crafts reaction) followed by hydrolysis and nitric acid oxidation (Scheme 9). The radical copolymerization of phosphonate-containing styrenic monomers leads to high molecular weight polymers [4].

**Scheme 10:**
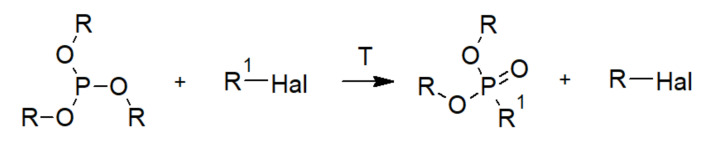
Synthesis of phosphonic acid compounds via Arbuzov reaction (R—alkyl, aryl etc., R’—alkyl, acyl etc., Hal—Cl, Br, I) [50].

Another possibility is immobilizing phosphonic acid ligands via the Arbuzov reaction (also known as Michaelis-Arbuzov rearrangement, Arbuzov rearrangement or Arbuzov transformation), which involves the reaction of an ester of trivalent phosphorus with alkyl halides resulting in the formation of carbon-phosphorus bond according to the following formula (Scheme 10) [49,50]:

This reaction originally discovered by Michaelis in 1898, then explored by Arbuzov, is one of the most versatile pathways for the formation of C-P bonds [50]. The Arbuzov reaction is commonly used for vinylbenzyl chloride-containing polymers. For example, Trochimczuk and Alexandratos [51] introduced phosphonic acid moieties into vinylbenzyl chloride/styrene copolymers. An Arbuzov reaction was followed by sulfonation resulting in bifunctional ion-exchange resins. The Arbuzov reaction was also used by Yu and co-workers [52] in the preparation of vinylbenzyl phosphonate diethyl ester (VBP) from vinylbenzyl chloride (VBC). Although this type of reaction was reported earlier [53,54], they found out that presented methods are not easily performable due to several issues, the most problematic of which is that required temperature condition (150 °C) leads to the VBC polymerization almost instantaneously. In fact, addition of some common nitrophenol inhibitors to the reaction mixture at lower temperature (ca. 100 °C) helps impede the spontaneous polymerization, but these compounds seem to degrade continuously and, additionally, cannot be easily removed from the final product. Yu et al. proposed a very effective inhibitor for the conversion of VBC to VBP: 6-*tert*-butyl-2,4-dimethylphenol. The obtained vinylbenzyl phosphonate diethyl ester can be easily polymerized by radical polymerization leading to colorless, transparent polymers [52].

**Scheme 11:**
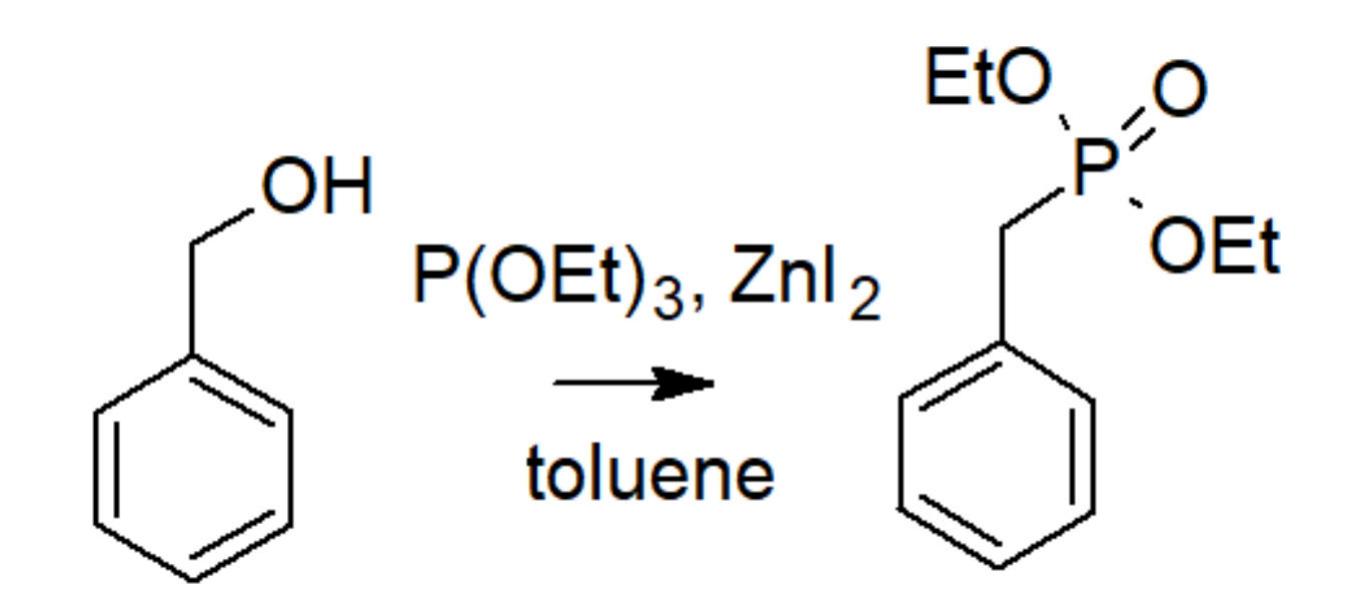
Schematic representation of phosphonate preparation from alcohols proposed by Barney et al. [55].

A few years ago, an interesting, alternative method for phosphonate preparation from alcohols using triethyl phosphite and zinc iodide was proposed by Barney et al. [55] (Scheme 11). This method might be used for polymer modification as an alternative to the Arbuzov reaction.

**Scheme 12:**
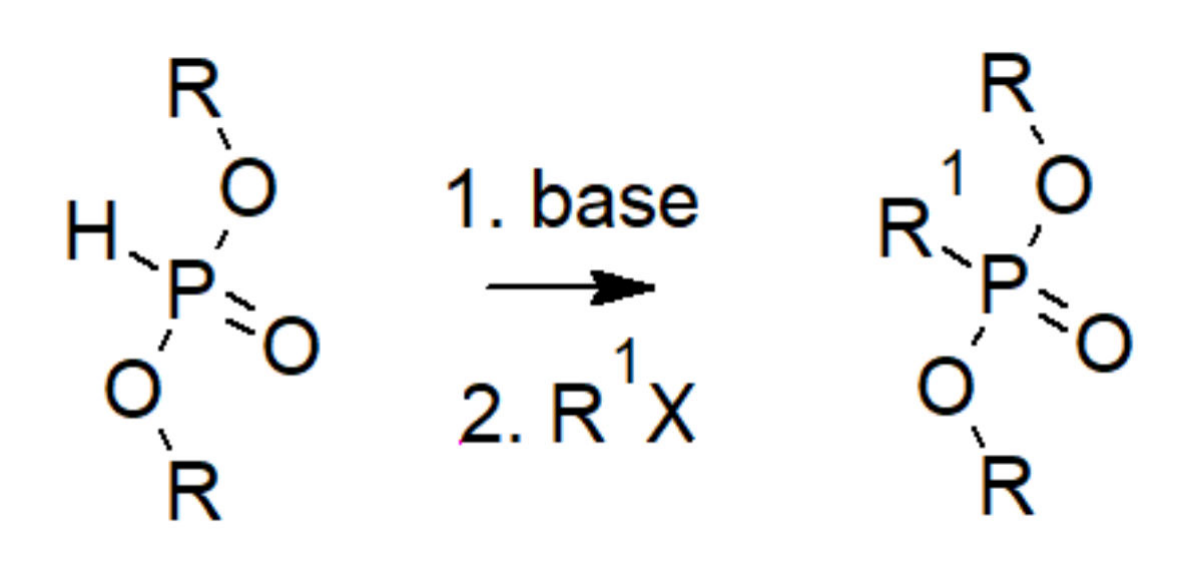
Synthesis of phosphonic acid compounds via Michaelis-Becker reaction.

Phosphonic acid moieties can also be incorporated into polymer backbones via the Michaelis−Becker reaction (Scheme 12), which is known to have several advantages over the conventional Arbuzov methodology. First of all, the Michaelis–Becker reaction proceeds rapidly at room temperature or below, providing high yields of products, which is very important in the case of vinyl reactants, because it prevents premature thermal polymerization. Furthermore, higher dialkyl-*p*-vinylbenzyl phosphonates, easily attainable by the Michaelis–Becker procedure, are difficult to obtain via the Arbuzov reaction [56].

**Scheme 13:**
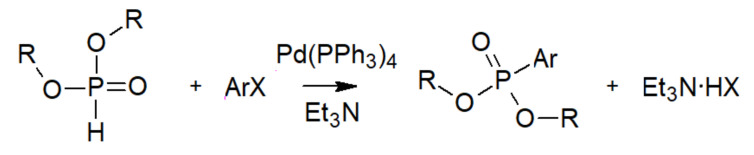
Cross-coupling reaction using palladium complex as a catalyst based on the example of aryl halide (X—Br, I) [62].

There are however few reports describing the usage of Michealis-Becker reaction in polymer science. Rajalakshmi et al. [57] incorporated Michaelis-Becker reaction into a two-step synthesis of dimethyl and diethyl 2-(methacryloyloxyethyl) phosphonates from 2-hydroxyethyl methacrylate (HEMA). Meziane et al. [58] proposed an efficient and rapid method for the synthesis of phosphonates or diphosphonates from various alkyl halides, based on the development of the Michaelis–Becker reaction under microwave irradiation. This approach led to better yields and shorter reaction time in comparison with classical heating technology. Moreover, less reactive fatty halides or secondary halides substrates may be used herein. The presented methodology was then successfully applied into the modification of polymer resin. Souzy et al. [59] synthesized a new perfluorovinyl ether monomer containing phosphonic acid functionalities using various phosphonation methods: Michaelis–Arbuzov, Michaelis–Becker organometallic techniques and palladium-catalysed arylation. It has been found that the type of synthetic route influences the yield and the best results were obtained when the reaction was catalyzed by a palladium complex. The use of palladium complexes as catalysts in the preparation of aryl- and vinyl phosphonates was first reported by Hirao et al. in the early 80s [60,61,62]. This method enables sp^2^ hybridized C-P bond formation (Scheme 13). Further modifications based on this approach, e.g., changes of catalyst, have been summarized in [60].

**Scheme 14:**
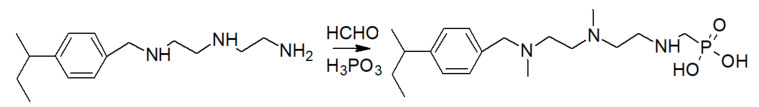
Mannich reaction based on the example of diethylenetriamine resin [63].

Alexandratos et al. incorporated phosphonic acid functionalities via the Mannich reaction (Scheme 14) [63]. The Mannich reaction is a very useful tool since it leads to the creation of a new C-C bond under mild conditions without complicated catalysts. However, when proper substrates are used, it can lead to creation of C-heteroatom bonds for incorporation of phosphorus-containing functionalities. This approach led to obtaining bifunctional polymer-supported aminomethyl-phosphonates whose Cd(II) complexating ability (98% Cd(II) and distribution coefficient of 2300) far exceed those of the monofunctional resins (ethylenediamine resin: 26% Cd(II), distribution coefficient of 26; phosphonic acid resin 49% Cd(II), distribution coefficient of 48). Some of the Mannich-type reactions are known by new names, e.g., the Kabachnik-Fields reaction (coupling of a carbonyl, an amine and a H-phosphonate compound resulting in obtaining α-aminophosphonates) [64] and the Moedritzer-Irani reaction (coupling of an amine, a formaldehyde and a phosphonic acid or its diesters resulting in *N*,*N*-disubstituted aminomethylphosphonic acids or *N*-substituted iminobis(methyl-phosphonic acids) [65].

**Scheme 15:**
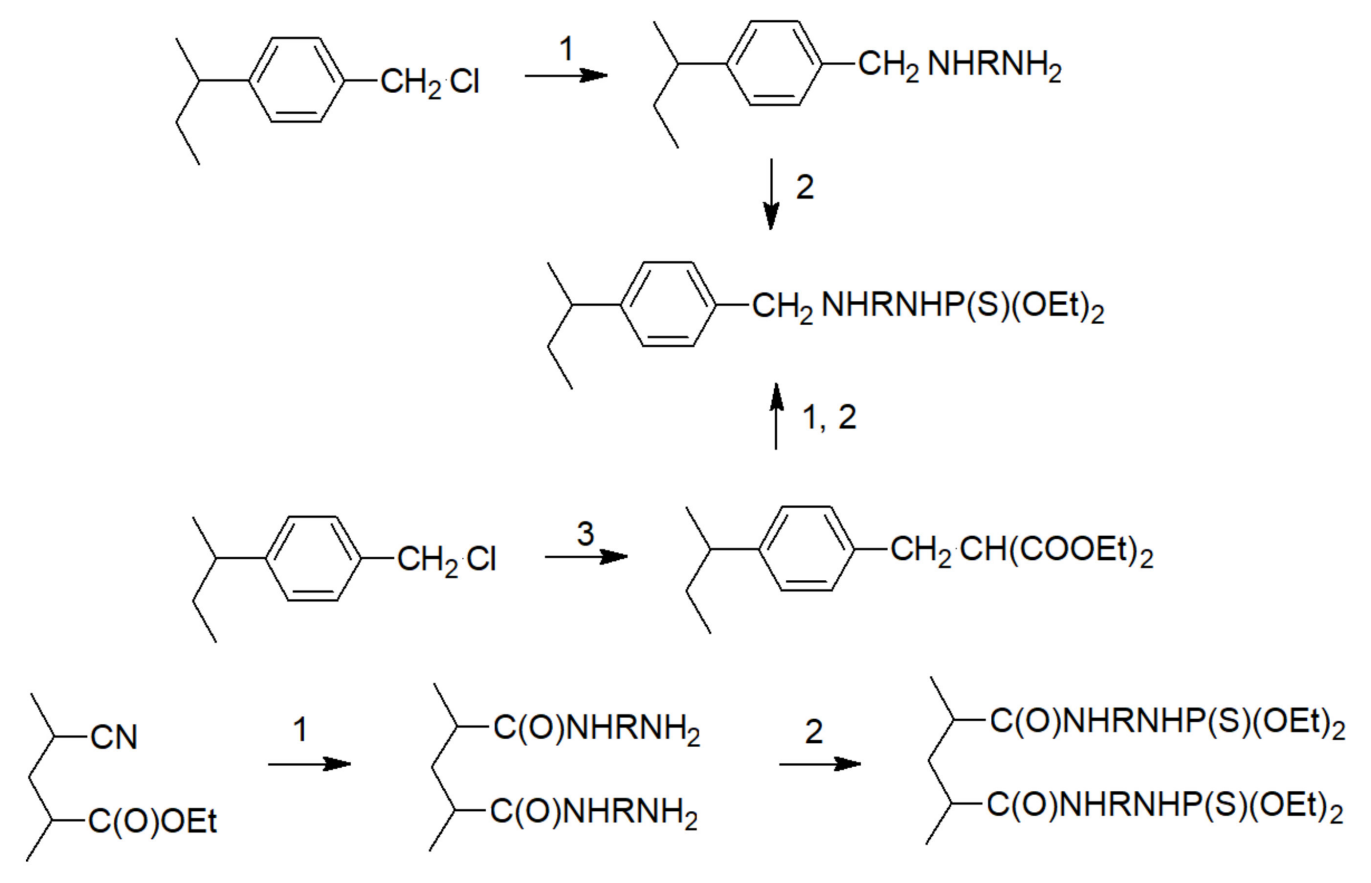
Schemes of aminothiophosphonate resins synthesis: 1—amine, 2—diethyl thiophosphonochloridate, 3—sodium diethyl malonate [66].

Chemical modification as a synthesis method for the preparation of chelating resins containing aminothiophoshponate functional groups was presented by Trochimczuk and Streat [66]. Such resins were able to separate trace levels of Cd(II) and Ni(II) in aqueous solutions. Synthesis routes were presented in Scheme 15.

It has been proven that aminothiophosphonate resin was highly selective toward Cd(II), while Ni(II) sorption was negligible. Moreover, the mechanism of metal adsorption was validated by comparison with a commercial resin—Purolite S950—containing aminomethylphosphonate ligands, and it was found to be predominantly coordination rather than ion exchange, whereas Purolite S950 interacts with metals by both of the mechanisms.

**Scheme 16:**
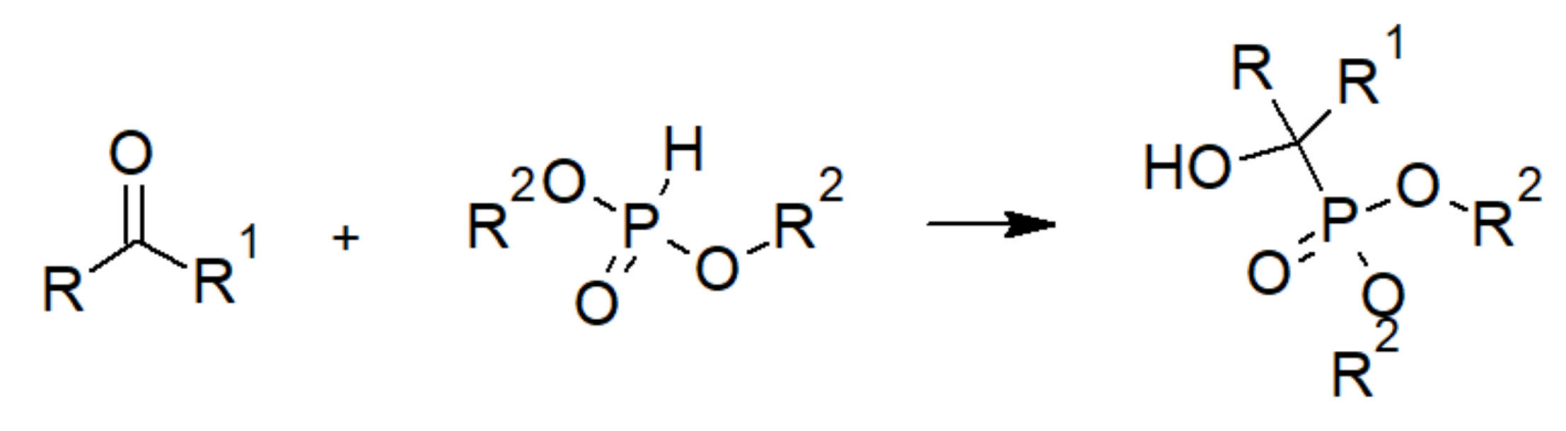
Schematic representation of Pudovik reaction (R, R^1^—H, alkyl, aryl, R^2^—alkyl, aryl) [67].

Polymer-supported phosphonic acids can be also prepared by the Pudovik reaction (Scheme 16). The Pudovik reaction involves the addition of compounds containing a labile P-H bond with unsaturated systems such as alkenes, carbonyls etc. and is one of the most versatile pathways for the formation of carbon-phosphorus bonds [9,67]. This approach has been used by Z. El Asri et al. in the preparation of phosphonated methacrylate - dimethyl(methacryloyloxy)-methyl phosphonate [9].

**Scheme 17:**
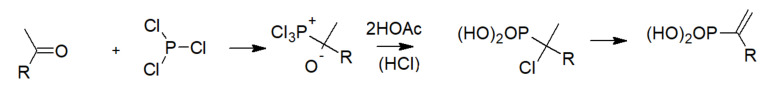
Synthesis of VPA monomer (1). R = H, alkyl group of 1–6 carbon atoms.

A polymer of great interest is poly(vinylphosphonic acid) (PVPA). There are a number of strategies for the preparation of its monomer—vinylphosphonic acid (VPA)—and some derivatives, described by Kabachnik and Medved [68]. One of the possible synthesis methods involves low cost precursors, such as phosphorus trichloride. The reaction is based on the nucleophilic addition of phosphorus trichloride followed by reaction with acetic acid and subsequent dehydrochlorination (Scheme 17) [69].

**Scheme 18:**
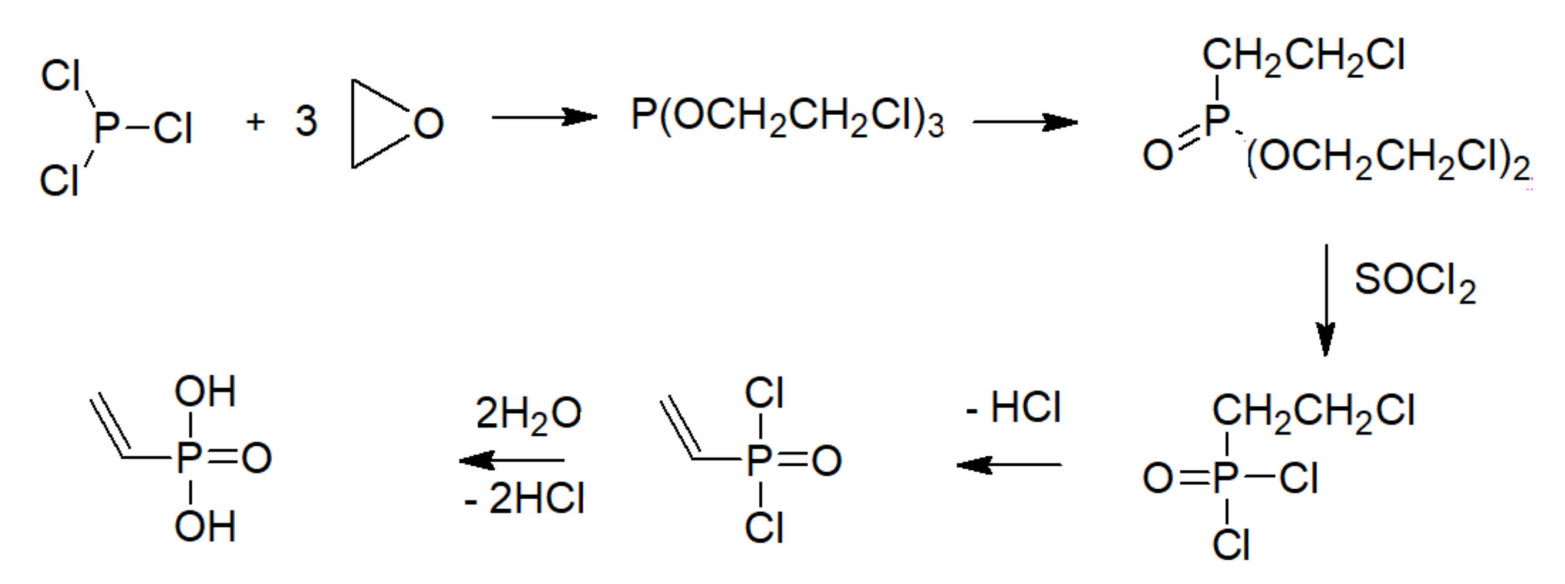
Synthesis of VPA monomer (2).

Another approach involves a 5-step reaction route presented in Scheme 18. In this method, phosphorus trichloride reacts with ethylene oxide resulting in tris(2-chloroethyl) phosphite, which is then rearranged to give bis(2-chloroethyl)-2-chloroethylphosphonate. The next step is the reaction of the bis(2-chloroethyl)-2-chloroethylphosphonate with thionyl chloride in the presence of triphenyl phosphine oxide acting as catalyst, to give 2-chloroethyl-phosphonic dichloride. Then 2-chloroethylphosphonic dichloride can be converted to vinylphosphonic acid by eliminating HCl in the presence of BaCl_2_ and the hydrolysis of the formed vinylphosphonic dichloride. However, this method requires a correspondingly high technical expenditure and the use of thionyl chloride which represents a risk involving step [69]. Syntheses of vinylphosphonic acid monomer, polymer and their derivatives have been described and summarized in [69].

### 3.3. Phosphinic Acid Polymers

**Scheme 19:**
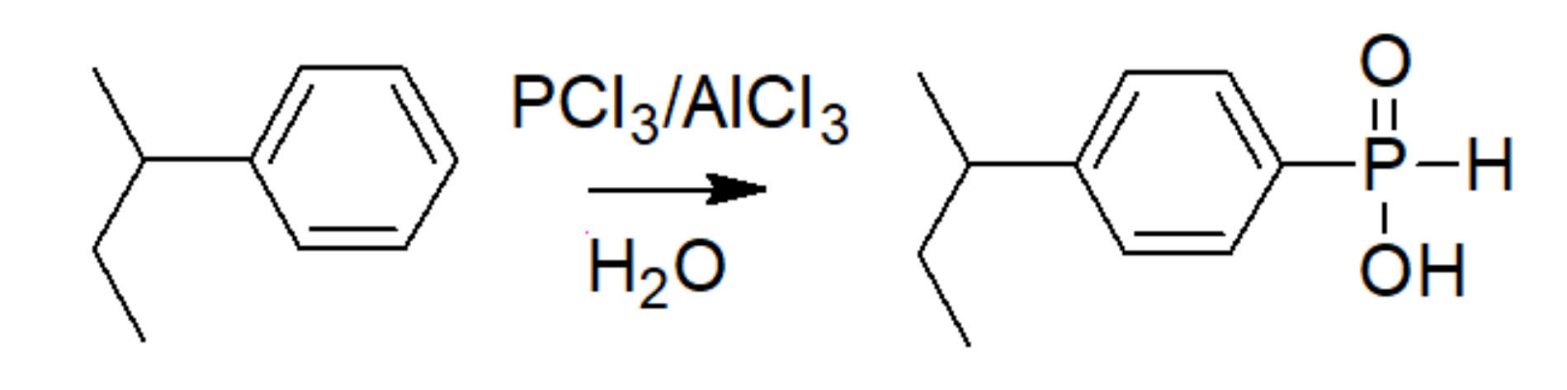
Polystyrene modification using phosphorus chloride and Friedel-Crafts catalyst.

Polymer-supported phosphinic acids have been studied i.a., by Alexandratos et al. [70]. Among the methods mentioned earlier, such resins can be prepared using Friedel-Crafts catalysts with PCl_3_ (Scheme 19). As reported by Alexandratos and co-workers, the order of catalyst activity is AlCl_3_ > FeCl_3_ > ZnCl_2_ > SnCl_4_, and, what is worth to mention, ferric chloride catalysis also operates by an additional redox mechanism which yields a phosphonic acid resin. Prepared resins exhibit dual-mechanism of ion exchange and metal-ion reduction [71].

**Scheme 20:**
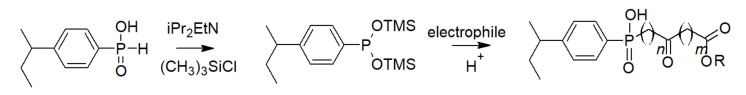
Synthesis route of the phenylphosphinic acid resin [72].

As reported by Trochimczuk, phenylphosphinic acid polymers obtained using Friedel-Crafts catalyst (Scheme 18) may also be employed as matrixes for the preparation of the multidentate ligand resins obtained through Michael reaction (Scheme 20). Three compounds were used as electrophiles: ethyloxalylchloride (*n* and *m* = 0), ethylbromopyruvate (*n* = 1, *m* = 0) and 4-chloroacetoacetate (*n* and *m* = 1). Such resins were able to selectively remove Au(III) in the presence of other metal chloro complexes [72].

## 4. Organophosphorus Polymers in Separation Science

### 4.1. Solvent Extraction and Organophosphorus Polymers in Separation Processes

The crucial issue to the success of metal separation is the choice of the ligand. Organophosphorus compounds have been widely used as complexating agents in solvent extraction due to their high distribution coefficients and high selectivity in addition to their low price. Such complexants demonstrate a high level of selectivity towards transition metals, lanthanides and actinides [4].

Solvent extraction consists of the removal of a targeted substance (one or more) from an aqueous phase to a so-called extractant forming a liquid organic phase. The extractant is composed of a complexant or extracting agent with or without solvent (diluent). The complexant should exhibit low solubility in the aqueous phase, chemical stability and must not form complexes with other compounds present in the aqueous phase (especially those more stable than targeted metal/ligand complexes). Furthermore, complexant is expected to be easily stripped of the loaded metals and relatively inexpensive [4,73]. The main advantages of solvent extraction include high throughput and available variety of complexants that have already been developed and tailored for numerous applications [4]. It also offers advantages of fast kinetics, high capacities, and selectivity towards target metal ions [74]. Taking disadvantages into account, one of the minor ones is the fact that the complexant may vanish through dissolution and lead to i.a., an entrainment by third layer formation as a result of incomplete phase separation [4]. Moreover, the finite aqueous solubility of the extractants, solvents and modifiers leads to the increase of the procedure costs, not to mention the possibility of water contamination with potentially toxic organics. Solvent extraction is not recommended for dilute solutions, as large volumes of extractant are needed [74].

In view of the aforementioned drawbacks of solvent extraction, it may thus be foreseen that combining phosphorus-containing ligands with crosslinked polymers—insoluble and easily separable from aqueous phase—is highly advantageous [4]. Ion exchange polymers operate on the same principles as solvent extraction [74]. Such solution eliminates extractant loss since it is covalently bonded to the polymer matrix. Furthermore, it is economical and environmentally friendly [4]. The resin may be regenerated and used many times and can be also used in continuous processes [74].

### 4.2. Developments in Organophosphorus Resins

Several stages may be distinguished when it comes to the development of phosphorus-containing resins. Undoubtedly the current market situation and historical events have a strong impact. To date, both economical and ecological reasons play significant roles in the recovery of metals. One of the problems encountered in the removal of metal ions is that the species of interest usually occurs in very low concentrations and exists in a complex matrix [74]. It is highly probable that benign ions present in this mixture (e.g., calcium, sodium etc.) will saturate the extractant before the target ion is effectively removed [74]. Thus, considerable efforts were made over the past decades for the preparation of more selective resins, especially since ion exchange was only one of the possible applications among e.g., ion-chromatography or catalysis [72].

Development of phosphonate ion-exchangers began in late 1940s, with phosphorylation of poly(vinyl alcohol) [75,76]. In 1952, Bregman et al. [77] reported the first known phosphorous and phosphonic cation exchange resins which show selectivity for sodium over potassium. The inversion of the order of selectivity for the alkali cations as compared to sulfonic resins was therein observed. Ion exchange resins with sulfonic ligands were exhaustively studied. However, based on the enthalpy of reaction of the sulfonic resins with alkali metal ions, Boyd et al. [78] reported in 1967 that the ion-exchange reaction alone does not have a wide enough range in the free energy of reaction to allow selectivity [79]. On the other hand, the carboxylic acid ligands, despite being more selective, require relatively high pH solutions to be effective [26], what is related with their pKa value (as stated in Section 2). The need for the polymers that are selective towards particular compounds was, and still is, one of the main challenges in the separation science field.

One of the major driving forces in the development of phosphorus-containing resins was the need to recover actinides and rare earth elements (REE). Several dozens of years of nuclear weapon and energy production has led to a significant amount of radioactive waste [80]. As described by Allard et al. [81], the production of actinides in the times of nuclear fission energy, as well as the nuclear fuel cycle, weapons testing, the use of radionuclide batteries etc. dramatically increased the risk of releasing actinides into the environment. The presence of those elements in ecosystems presents a major health hazard. Thus, it was necessary to develop technologies enabling an effective separation of radioactive elements. Moreover, global warming observed in recent years in combination with burning of hydrocarbon-based fuels has placed renewed emphasis on alternative sources of energy. The essential solution to public and political acceptance of nuclear energy lies in the implementation of efficient separations throughout the nuclear fuel cycle [82,83]. A thorough review on the methods for uranium removal has been provided by Aly and Hamza [84]. Solvent extraction is the most commonly used process-scale separation technique for nuclear applications and phosphorus-containing extractants such as tributylphosphate (TBP) are favorably used [85]. TBP has been used in the separation of uranium, thorium and plutonium since the Manhattan Project in World War II. However, such systems are deployed in highly radioactive environments, and the radiation chemistry as well as hydrolytic stability of the ligands play a significant role in determining extraction efficiency, separation factors etc. [86]. Along the years, many phosphorus-containing resins were examined for uranium recovery from different media, e.g., Diphonix^®^, RSPO, Diaion-CRP200, Purolite S940, Lewatit OC1060, Actinide-CU [86,87,88,89,90]. Synthetic polymers have attracted the most interest for the recovery of uranium from seawater [91]. The review on this topic was provided by Kim et al. [91].

REEs refers to fifteen metallic elements of the lanthanide series, coupled with the chemically similar yttrium, and, occasionally, scandium [92]. REE are rather abundant in the earth’s crust in comparison to other commonly exploited elements, but are not sufficiently concentrated to be easily exploitable. They are very difficult to separate mostly due to the similarity in their ionic radii. However, they possess unique properties, what expands their application in many fields, especially high-tech components, green technologies, material industries of high-temperature superconductors, secondary batteries, hybrid cars and many others [93].

In the 1950s, South Africa, India and Brazil had rare earth mines in operation. During the 1960s to 1980s, the Mountain Pass in California became the largest global producer and that lasted till its closure in 2002. In 1990s China began large scale production and currently is the largest worldwide producer of rare earth elements, accounting for ~97% of the total world supply [93,94]. However, the Chinese government has drastically limited the export of rare earths up to 35,000 tons, much below the yearly demand of other countries. Before 1960, the ion exchange method was the only way to separate rare earth metals [93]. Separation of rare earth metals employing ion exchange processes was originally initiated to separate fission products obtained from nuclear reactors. In 1964, Powell wrote that the development of ion-exchange processes for the separation of REE was prompted by a need: (1) to separate small amounts of REE from each other for mixtures analyses, and (2) to separate huge amounts of highly purified REEs for research and technology purposes [95]. In 1987, Alexandratos et. al. confirmed that phosphinic resin complexes more lanthanides and actinides(Eu(III), Am(III), UO_2_(II) and Pu(IV)) than the sulfonic resin from acidic solutions [26]. Nowadays, the recovery of REE from various resources is one of the most important topics when it comes to phosphorus-based resins.

An interesting group of resins that combines the inherent advantages of both liquid/liquid extraction and ion exchange separation are solvent-impregnated resins (SIRs) [96]. The term “impregnation” was originally introduced to the ion-exchange literature by Lederer in 1958 [97]. Relatively simple and versatile synthesis of SIRs led to obtaining of a whole range of materials tailored to particular ions and presents an opportunity to use various combinations of polymeric supports and ionic liquid extractants to achieve that [96]. The employed extractants can exhibit strong affinity for the polymeric matrix but still act as if in the liquid phase [98]. The main advantages of SIR technology are the higher adsorption capacity and easier regeneration when compared to classical adsorbents for organics removal as well as flexibility with respect to applications and the fact that the right complexing agents in the precise amount are easily introduced during the impregnation process. The biggest disadvantage of SIRs is the loss of the impregnated extractant due to its solubility in the aqueous phase that leads to a steady loss of adsorptive capacity, rendering the SIRs ineffective after several cycles of application. As the leakage is likely to contain toxic and odorous compounds, this problem cannot be discounted from an environmental point of view and is probably the main reason why SIR technology has not evolved into large-scale application [96]. Detailed information about SAPs has been provided in the review prepared by Kabay and co-workers [96]. The preparation methods and physical properties of SIRs containing acidic organophosphorus extractants were reviewed by Juang [98].

**Figure 1:**
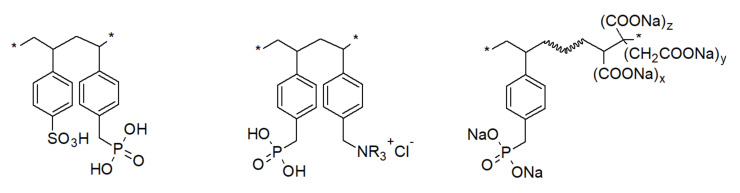
From the left: resin with phosphonic and sulfonic functional groups [51], phosphonic and amino groups [99], phosphonic and various carboxylic groups [100].

In the 1980s, among many types of ion-exchangers possessing acidic ligands, those possessing at least two types of functional groups gained particular attention [72]. Two or more functional groups can cooperate with each other in the process of metal ions separation. First, this property was used in liquid-liquid extraction. However, placing two or more different functional groups on a polymer matrix is a good way to solve problems with their proximity—as explained by Trochimczuk, all factors reducing mobility and increasing the distance between functional groups have adverse effect on ion removal [72]. Many works describing synergistic properties of different functional groups were published over the years [72]—e.g., resins with phosphonic and amino groups [99], phosphinic [70], phosphonic and sulfonic [51], phosphonic and various carboxylic groups [100]. For instance, in highly acidic conditions or in the presence of excess of sodium ions, sulphonic groups contributed only to hydrophilicity of the resin, thus increasing transport of ions, whereas phosphonic ones were responsible for the resins’ selectivity [51]. The presence of bulk ions, such as sodium or calcium, is one of the major problems in the removal of trace metals. Exemplary structures of bifunctional resins are presented in Figure 1.

Several classes of Dual Mechanism Bifunctional Polymers (DMBPs) may be distinguished [26]:

- class I—ion exchange/redox resins, e.g., phosphinic acid resin is capable of ion exchange with any metal ion, but also of reducing the limited number of ions such as Hg (II), Ag (I), Cu (II), Pd(II) to free metal, thus oxidizing the ligand to phosphonic acid;
- class II—ion exchange/coordination resins; DMBPs composed of phosphonic acid (access mechanism) and coordinating ligand (amine, ester—recognition mechanism), e.g., phosphonate diester/monoester resin, where a synergistic enhancement in the amount of complexed Ag(I) in comparison to corresponding monofunctional resin was observed;
- class III—ion exchange/precipitation resins; e.g., resin composed of phosphonic acid ligands and quaternary ammonium ligand. The former ligand allows ions into the polymer network, the latter carrying an associated anion leads to the formation of insoluble salt when a given cation comes into close proximity. Process occurs rapidly and the precipitate is retained within polymer bead.

Such systems have been widely studied by Alexandratos et al. [6]. DMBPs classes and its sorption capacity towards different ions have been summarized in [4]. Among DMBPs, Alexandratos and co-workers have extensively studied phosphorus-containing polymers as ion-exchange resins [6,41,51,63,70,71,79,83,101,102,103,104,105,106,107,108,109,110,111,112,113].

**Scheme 21:**
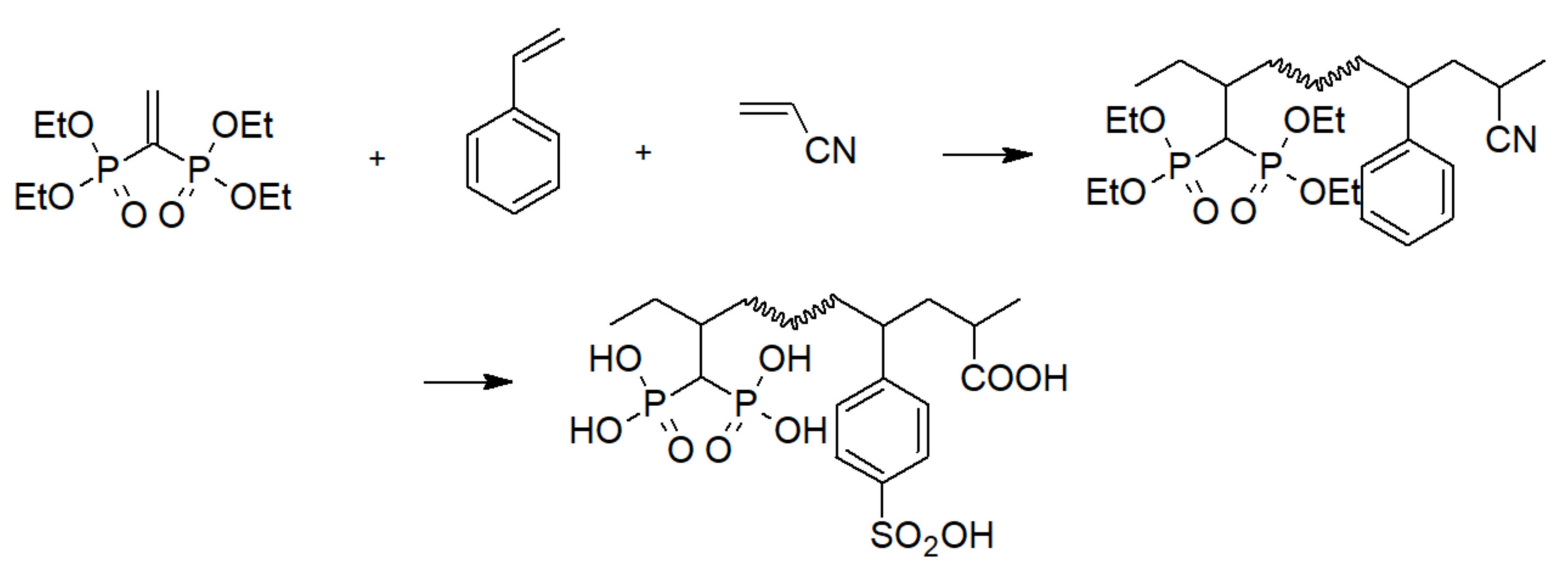
Synthesis of Diphonix^®^ [4].

Diphosphonic acid polymers are widely used since they possess the ability to extract U(VI) Pu(IV), Np(IV), Am(III) Eu(III), Th(IV) and some transition metals. It is known that compounds with such ligands have very high stability constants with metal ions [107]. For example, these resins exhibit far greater rates of complexation of Eu(III) and are able to complex metal ions from highly acidic solutions through diphosphoryl ligand [4]. One of the most popular commercially available diphosphonic acid resin is Diphonix^®^, developed by the Argonne National Laboratory and the University of Tennessee. Synthesis of Diphonix^®^ has been shown in the Scheme 21 [4]. The preparation process involves copolymerization of tetraalkylvinylidene diphosphonate with styrene and acrylonitrile followed by sulphonation with concentrated sulphuric acid. The presence of carboxylic and sulphonic groups determines better hydrophylic properties [114]. Diphosphonic acid polymers can also be obtained via chemical modification of poly(vinylbenzyl chloride) [104].

Diphonix^®^ can be used in low pH and is characterized by high affinity towards U(VI), Pu(IV), Np(IV), Th(IV), Am(III), Eu(III). Its performance is much more efficient when compared to other resins. Detailed information is summarized elsewhere [114].

**Scheme 22:**
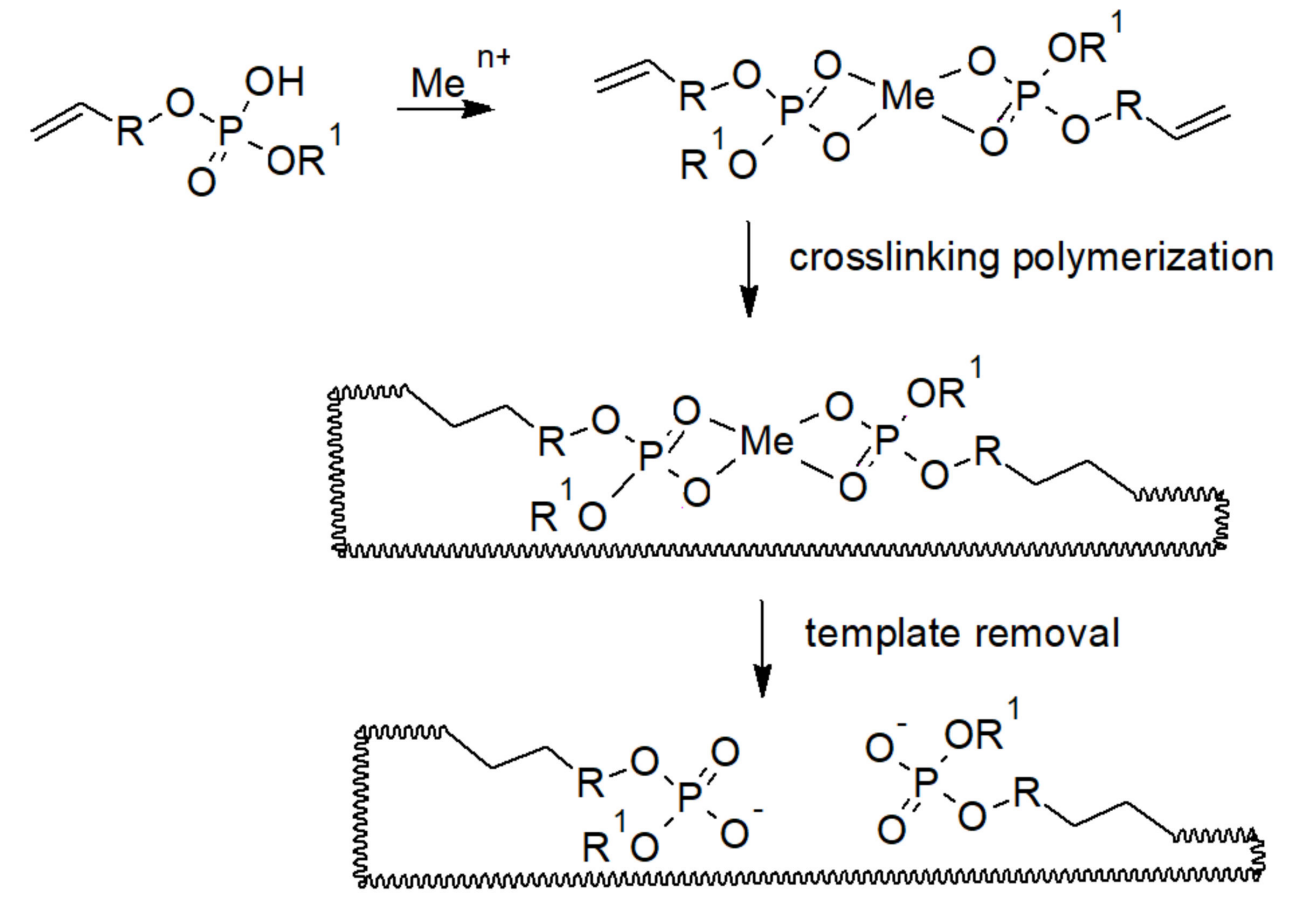
Schematic representation of molecular imprinting using phosphoric acid monomer (Me^n+^—metal ion).

Another approach to obtain effective and selective ion-exchange resins is molecular imprinting (Scheme 22) [4,115,116]. Molecularly imprinted polymers (MIPs) are prepared by contacting template species such as molecules, ions etc. to be extracted in subsequent application and monomer with phosphorus-containing ligands. The second step is polymerization with a crosslinking agent to obtain a three-dimensional network. The last part requires removal of the template resulting in the formation of cavities complementary in shape, size and functionality to particular species. Unfortunately, the need for a high crosslink level leads to a diminished loading capacity measured in micromoles/gram [4].

Detailed factsheets containing absorption capacities of different phosphorus-containing resins toward metal ions have been provided elsewhere [4,32]. However, selected data originating from the articles cited in this review has been collected in Table 2.

## 5. Summary

High distribution coefficients and selectivity, together with relatively low price promote the use of organophosphorus compounds as complexating agents in separation processes. Due to the disadvantages associated with solvent extraction (i.a., extractant loss, procedure costs, environmental issues etc.), polymer resins—operating on the same principles, but insoluble and easily separable from aqueous phase—have received a lot of attention in the past few decades and remain the center of interest for many research groups. The uniqueness of polymers described herein arises, e.g., from their ionization potential. Above their pKa, they are able to bind metals through an ion-exchange mechanism, whereas below it—through complexation. This feature leads to their better selectivity when compared with strong acid polymers. They are proved to be selective especially toward lanthanides, U(VI), Ti(IV) and Fe(III). This review ventures to describe the preparation and the most important properties of phosphoric, phosphonic and phosphinic acid polymers applied in separation processes, as well as highlights the most important achievements in the field. However, one should note such materials are already effectively applied or extend their potential to the use in many other fields, such as catalysis, biological and proton conducting membranes for fuel cells, flame-retardancy, anticorrosion systems, tissue engineering or drug delivery [5]. Undoubtedly, many more are bound to be developed in the near future.

## Figures and Tables

**Table 1:** Bond energies of chosen phosphorus-containing linkages [3].

Bond	Bond Energy [kJ/mol]
P-H	322
P-P	209
P-C	272
P-O	360
P=O	586
P-N	230
P=C	578
P≡P	490
P=N	687
P-Cl	321
P-F	527
P-I	184
P-Br	264

**Table 2:** The application of organophosphorus resin in separation processes—a collection of chosen papers.

P-Containing Functional Group	Polymer	Application/Features	References
‘Phosphoric acid	Cellulose phosphate	Sorption from 4N-acid: high affinity for Th^4+^, Ti^4+^, U^4+^, Ce^4+^, Fe^3+^, ZrO^2+^, UO_2_^2+^	[29]
		Cation exchanger in calcium-hydrogen cycle	[30]
		Adsorption of proteins	[33]
	Poly(glycidyl methacrylate-*co*-divinylbenzene) functionalized with phosphoric acid	Selective resins (lithium adsorbed in preference to sodium and potassium, better adsorption of hard-Lewis cations from acidic media, highest selectivity towards lead)	[38]
	Poly(glycidyl methacrylate-*co*-divinylbenzene) and poly(glycidyl methacrylate) functionalized with phosphoric acid or phosphorus oxychloride	Resins with high cation exchange capacity, salt splitting capacity, and adsorption capacity for Cu^2+^, Zn^2+^, Cd^2+^, Ca^2+^, and Ag^+^; RGP obtained from poly(glycidyl methacrylate) – high adsorption capacity for Al^3+^, Fe^3+^, and UO_2_^2+;^ phosphoric acid functionalization – higher selective adsorption for Li^+^ than for Na^+^	[40]
Phosphonic acid	D2EHPA/TOPO impregnated resins, ion exchange resin containing aminophosphonic acid groups	Uranium removal – D2EHPA/TOPO resins are not affected by the presence of Fe^2+^, which reduces uranium to the tetravalent oxidation state, prevents uranium sorption.	[89,90]
	α-, β -, and γ-ketophosphonate polymer-supported resins	Sorption of metal ions (Eu(III), Cu(II), Pb(II), Cd(II), Co(II), Ag(II)) from nitric acid	[41]
	Bifunctional ion-exchange resins synthesized from vinylbenzyl chloride– styrene copolymers: Arbuzov reaction followed by sulfonation	Eu(III) sorption from acidic solutions	[51]
	Non-ionic phosphorylated resin diethyl polystyrene-methylenephosphonate (EPMP)	Selective adsorption of hexavalent uranium	[54]
	Bifunctional aminomethylphosphonic acid resins (Mannich reaction)	Sorption of Cu(II), Cd(II), Pb(II), and Eu(III)—cadmium ion complexation shows the greatest bifunctional effect,	[63]
	Ion-exchange/chelating resins with aminomethylphosphonate groups (Fields-Kabatschnik reaction)	The removal of Cu(II), Cd(II), Ni(II), and Zn(II)	[64]
	Diphonix^®^	Uranium removal (after 2h—20.7%, 24h—18.0% from 40% H_3_PO_4_)	[68]
		Actinides ion removal from very acidic solutions	[101]
		Uptake of various transition and post-transition metal ions; a very high affinity for Fe(III) and Cr(III) in very acidic solutions	[113]
		Gallium(III) and indium(III) recovery	[105]
		Chelating diphosphonic acid groups are grafted to a silica support to overcome some of the problems associated with the stripping of actinide elements from the resin (Diphosil)	[106]
		Diphonix A – additional strong base anion exchange groups; the uptake of Se(IV), tri-, tetra- and hexa-valent actinides and pertechnetate anions	[108]
		The of uptake of several actinide ions [Am(III), U(VI), Th(IV), Np(IV) and Pu(IV)] and of some transition-metal ions [Co(II), Zn(II), Fe(III) and Cr(III)] at tracer concentration level;	[111]
	Bifunctional resin consisting of phosphonic acid and quaternary amine groups	Sorption of barium, calcium, silver cations; changing the associated anion allows for control of the ionic recognition process	[99]
	Poly(vinylbenzyl chloride-*co*-styrene-*co-*divinylbenzene) functionalized with the sodium salt of tetra(isopropyl) methylene diphosphonate	Eu(III) sorption; highly selective resins with rapid complexation kinetics: 99.7% Eu(III) complexed from a 1M nitric acid solution with a 30-min contact time	[104]
Diphosphonate	Chelating resin with diphosphonate groups synthesized from the tetraethylester of 1,1-vinylidenediphosphonic acid	Eu(III) sorption: sulfonic acid ion exchange resin complexes 14.3% Eu(III), diphosphonate-based polymer complexes 96.5% from a 1N HNO_3_/0.4N NaNO_3_ solution	[107]
Phosphate and phosphonate diesters	Polymer-supported phosphonate and phosphate diester ligands	The complexation of uranium(VI) e.g., the expanded gel resin - phosphorylated pentaerythritol: 99% complexation within 20 min	[83]
	Polystyrene-divinylbenzene matrix with methylenediphosphonate, carboxylate, and sulfonate functional groups	Exceptionally high affinity for polyvalent cations even from moderately acidic aqueous media	[110]
Phosphonic acid, phosphonate ester	Resins with phosphonic acid/phosphonate ester ligands and phosphonic acid/tertiary amine ligand on PS support	Americum extraction	[79]
Phosphonates, phosphinic acid	Bifunctional polymer resins (I DMBP, II DMBP, compared with monofunctional resins)	Metal-specific reactions, polymers with high level of ionic recognition; phosphinic ligands—ability to control the recognition mechanism through the reduction reaction with Hg(II); phosphonic resin—sorption of Ag, Hg and Fe in different conditions—high complexation of Fe; complexation of Fe is adversely affected by the presence of diester ligands	[102]
Phosphinic acid	Bifunctional phosphinic acid ion-exchange/redox resins	Dual mechanism sorption (ion-exchange and metal-ion reduction) of Ag(I) and Hg(II): after a 9-h contact time with Ag(I) ions and a 2-h contact time with Hg(II) ions all of the primary acid sites are oxidized	[71]
	Phenylphosphinic acid resins (Michael reaction)	the removal of Cu(II), Cd(II), Ni(II), Zn(II), Mg(II) and Ca(II) from nitric acid solutions	[72,76]
Dihydroxyphosphino- and/or phosphono groups	The macroreticular chelating resins containing dihydroxy-phosphino and/or phosphono groups	Recovery of uranium from sea water—e.g., the average recovery ratios of uranium from sea water with Na-form and H-form RCSP on 10 recycles: 84.9% and 90.5%, respectively	[86,87]

## References

[1] Hanson P.R. (2014). Organophosphorus chemistry. Beilstein J. Org. Chem..

[2] Quin L.D. (2000). A Guide to Organophosphorus Chemistry.

[3] Maiti S., Banerjee S., Palit S.R. (1993). Phosphorus-containing polymers. Prog. Polym. Sci..

[4] Ripperger K.P., Alexandratos S.D. (1999). Polymer-Supported Phosphorus-Containing Ligands for Selective Metal Ion Complexation. Adsorption and Its Application in Industry and Environmental Protection Vol II: Appliactions in Environmental Protecion.

[5] Monge S., David G. (2014). Phosphorus-Based Polymers–From Synthesis to Applications.

[6] Yang Y., Alexandratos S.D. (2009). Affinity of Polymer-Supported Reagents for Lanthanides as a Function of Donor Atom Polarizability. Ind. Eng. Chem. Res..

[7] Alexandratos S.D., Crick D.W. (1996). Polymer-Supported Reagents: Application to Separation Science. Ind. Eng. Chem. Res..

[8] Janauer G.E., Ramseyer G.O., Lin J.W. (1974). Selective Separations by Reactive Ion Exchange with Common Polystyrene-Type Resins. Part, I. General Considerations. Anal. Chim. Acta.

[9] El Asri Z., Chougrani K., Negrell-Guirao C., David G., Boutevin B., Loubat C. (2008). An Efficient Process for Synthesizing and Hydrolyzing a Phosphonated Methacrylate: Investigation of the Adhesive and Anticorrosive Properties. J. Polym. Sci. A1 Polym. Chem..

[10] Markova D., Opper K.L., Wagner M., Klapper M., Wagener K.B., Mullen K. (2013). Synthesis of proton conducting phosphonic acid-functionalized polyolefins by the combination of ATRP and ADMET. Polym. Chem..

[11] David G., Negrell C., Manseri A., Boutevin B. (2009). Poly(MMA)-b-poly(monophosphonic acrylate) Diblock Copolymers Obtained by ATRP and Used as Additives for Anticorrosive Coatings. J. Appl. Polym. Sci..

[12] Barthélémy B., Devillers S., Minet I., Delhalle J., Mekhalif Z. (2011). Surface-initiated ATRP of 2-(methacryloyloxy)ethyl 2-(trimethylammonio)ethyl phosphate on Phynox. Appl. Surf. Sci..

[13] Barnard Z.Z., Keen I., David J.T.H., Traian V., Chirila D., Harkin G. (2008). PHEMA Hydrogels Modified through the Grafting of Phosphate Groups by ATRP Support the Attachment and Growth of Human Corneal Epithelial Cells. J. Biomater. Appl..

[14] Yuan B., Chen Q., Ding W.-Q., Liu P.-S., Wu S.-S., Lin S.-C., Shen J., Gai Y. (2012). Copolymer Coatings Consisting of 2-Methacryloyloxyethyl Phosphorylcholine and 3-Methacryloxypropyl Trimethoxysilane via ATRP To Improve Cellulose Biocompatibility. Appl. Mater. Interfaces.

[15] Zhou F., Huck W.T.S. (2005). Three-stage switching of surface wetting using phosphate-bearing polymer brushes. Chem. Commun..

[16] Yu X., Yang X., Horte S., Kizhakkedathu J.N., Brooks D.E. (2013). ATRP synthesis of poly(2-(methacryloyloxy)ethyl choline phosphate): A multivalent universal biomembrane adhesive. Chem. Commun..

[17] Huang J., Matyjaszewski K. (2005). Atom Transfer Radical Polymerization of Dimethyl(1-ethoxycarbonyl)vinyl Phosphate and Corresponding Block Copolymers. Macromolecules.

[18] Ma Y., Tang Y.N.C., Armes S.P., Lewis A.L., Lloyd A.W., Salvage J.P. (2003). Well-Defined Biocompatible Block Copolymers via Atom Transfer Radical Polymerization of 2-Methacryloyloxyethyl Phosphorylcholine in Protic Media. Macromolecules.

[19] Canniccioni B., Monge S., David G., Robin J.J. (2013). RAFT polymerization of dimethyl(methacryloyloxy)-methyl phosphonate and its phosphonic acid derivative: A new opportunity for phosphorus-based materials. Polym. Chem..

[20] David G., El Asri Z., Rich S., Castignolles P., Guillaneuf Y., Lacroix-Desmazes P., Boutevin B. (2009). Peculiar Behavior of Degenerative Chain Transfer Polymerization of a Phosphonated Methacrylate. Macromol. Chem. Phys..

[21] Baratha K.V., Nourry A., Pilard J.-F. (2015). Synthesis of NR based polyurethanes containing phosphorylated polymers as chain extenders. Eur. Polym. J..

[22] Yusa S., Fukuda K., Yamamoto T., Ishihara K., Morishima Y. (2005). Synthesis of Well-Defined Amphiphilic Block Copolymers Having Phospholipid Polymer Sequences as a Novel Biocompatible Polymer Micelle Reagent. Biomacromolecules.

[23] Stenzel M.H., Barner-Kowollik C., Davis T.P., Dalton H.M. (2004). Amphiphilic Block Copolymers Based on Poly(2-acryloyloxyethyl phosphorylcholine) Prepared via RAFT Polymerisation as Biocompatible Nanocontainers. Macromol. Biosci..

[24] Bhuchar N., Deng Z., Ishihara K., Narain R. (2011). Detailed study of the reversible addition–fragmentation chain transfer polymerization and co-polymerization of 2-methacryloyloxyethyl phosphorylcholine. Polym. Chem..

[25] Uchiyama M., Sato K., Kamigaito M. (2016). A phosphonium intermediate for cationic RAFT polymerization. Polym. Chem..

[26] Alexandratos S.D. (1992). Polymer-Supported Reagents with Enhanced Metal Ion Recognition: Application to Separations Science. Separ. Purif. Method..

[27] Alexandratos S.D., Hussain L.A. (1996). Bifunctionality as a Means of Enhancing Complexation Kinetics in Selective Ion Exchange Resins. Ind. Eng. Chem. Res..

[28] Jyo A., Jamabe K., Kimura G. (1999). Properties of phosphoric acid resins derived from poly(glycidyl methacrylates) crosslinked with dimethacrylates of oligo(ethylene glycols), Special Publication. R. Soc. Chem..

[29] Head A.J., Kember N.F., Miller R.P., Wells R.A. (1958). Ion-exchange properties of cellulose phosphate. J. Chem. Soc..

[30] Jurgens J.F., Reid D., Guthrie J.D. (1948). Phosphorylated Cotton Cellulose as a Cation-Exchange Material. Text. Res. J..

[31] Hoffpauir C.L., Guthrie J.D. (1950). Ion-Exchange Characteristics of Chemically Modified Cotton Fabrics. Text. Res. J..

[32] Guthrie J.D. (1952). Ion Exchange Cottons. Ind. Eng. Chem..

[33] Peterson E.A., Sober H.A. (1956). Chromatography of Proteins. I. Cellulose Ion-exchange Adsorbents. J. Am. Chem..

[34] Reid J.D., Mazzeno L.W. (1949). Preparation and Properties of Cellulose Phosphates. Ind. Eng. Chem..

[35] Ford F.M., Hall W.P. (1949). Flameproofing of Fibrous Materials. U.S. Patent.

[36] Jayakumar R., Reis R.L., Mano J.F. (2006). Chemistry and Applications of Phosphorylated Chitin and Chitosan. e-Polymers.

[37] Furukawa J., Kobayashi E., Wakui T. (1980). Preparations and Chelating Properties of Ethyl-(p-vinylphenyl)-phosphoric Acid or Ethyl-(p-vinylphenyl)thiophosphoric-Acid Polymer and Copolymer. Polymer. J..

[38] Jyo A., Matsufune S., Ono H., Egawa H. (1997). Preparation of phosphoric acid resins with large cation exchange capacities from Macroreticular poly(glycidyl methacrylate-co-divinylbenzene) beads and their behavior in uptake of metal ions. J. Appl. Polym. Sci..

[39] Jyo A., Zhu X., Pawłowski L., Gonzales M.A., Dudzińska M.R., Lacy W.J. (1998). Metal-Ion Selectivity of Phosphoric Acid Resin in Aqueous Nitric Acid Media. Chemistry for the Protection of the Environment 3.

[40] Egawa H., Nonaka T., Maeda H. (1985). Studies on selective adsorption resins. XXI. Preparation and properties of macroreticular chelating ion exchange resins containing phosphoric acid groups. J. Appl. Polym. Sci..

[41] Alexandratos S.D., Hussain L.A. (1998). Synthesis of α-, β-, and γ-Ketophosphonate Polymer-Supported Reagents: The Role of Intra-ligand Cooperation in the Complexation of Metal Ions. Macromolecules.

[42] Klee J.E., Lehmann U. (2010). Novel 2-(ω-phosphonooxy-2-oxaalkyl)acrylate monomers for self-etching self-priming one part adhesive. Beilstein J. Org. Chem..

[43] Moszner N., Salz U., Zimmermann J. (2005). Chemical aspects of self-etching enamel–dentin adhesives: A systematic review. Dent. Mater..

[44] Salz U., Zimmermann J., Zeuner F., Moszner N. (2005). Hydrolytic stability of self-etching adhesive systems. J. Adhes. Dent..

[45] Moszner N., Zeuner F., Pfeiffer S., Schurte I., Rheinberger V., Drache M. (2001). Monomers for Adhesive Polymers, 3 - Synthesis, Radical Polymerization and Adhesive Properties of Hydrolytically Stable Phosphonic Acid Monomers. Macromol. Mater. Eng..

[46] Sahin G., Albayrak A.Z., Bilgici Z.S., Avci D. (2009). Synthesis and Evaluation of New Dental Monomers with Both Phosphonic and Carboxylic Acid Functional Groups. J. Polym. Sci. Part A.

[47] David G., Boutevin B., Seabrook S., Destarac M., Woodward G., Otter G. (2007). Radical Telomerisation of Vinyl Phosphonic Acid with a Series of Chain Transfer Agents. Macromol. Chem. Phys..

[48] David G., Boyer C., Tayouo R., Seabrook S., Ameduri B., Boutevin B., Woodward G., Destarac M. (2008). A Process for Polymerizing Vinyl Phosphonic Acid with C_6_F_13_I Perfluoroalkyl Iodide Chain-Transfer Agent. Macromol. Chem. Phys..

[49] Michaelis A., Arbuzov B.A. (1906). Reactions of alkyl halides with phosphites. J. Russ. Phys. Chem. Soc..

[50] Bhattacharya A.K., Thyagarajan O. (1981). The Michaelis-Arbuzov Rearrangement. Chem. Rev..

[51] Trochimczuk A.W., Alexandratos S.D. (1994). Synthesis of bifunctional ion-exchange resins through the Arbusov reaction: Effect on selectivity and kinetics. J. Appl. Polym. Sci..

[52] Yu Z., Zhu W.X., Cabasso I. (1990). Synthesis and polymerization of vinylbenzylphosphonate diethyl ester. J. Polym. Sci. Part A.

[53] Cabasso I., Jagur-Grodzinski J., Vofsi D. (1974). Synthesis and Characterization of Polymers with Pendent Phosphonate Groups. J. Appl. Polym. Sci..

[54] Kennedy J., Burford F.A., Sammes P.G. (1960). The selective adosption of hexavalent uranium by a non-ionic phosphorylated resin from solutions of di-normal-butylphosphoric acid in benzene. J. Inorg. Nucl. Chem..

[55] Barney R.J., Richardson R.M., Wiemer D.F. (2011). Direct Conversion of Benzylic and Allylic Alcohols to Phosphonates. J. Org. Chem..

[56] Wyman P., Crook V.L., Hunt B.J., Ebdon J.R. (2004). Improved synthesis of phosphorus-containing styrenic monomers. Des. Monomers Polym..

[57] Rajalakshmi K., Santhana Gopala Krishnan P., Nayak S.K. (2015). Synthesis of Dialkyl 2-(Methacryloyloxyethyl) Phosphonates, Their Characterization and Polymerization. Polym. Sci. Ser. B.

[58] Meziane D., Hardouin J., Elias A., Guénin E., Lecouvey M. (2009). Microwave Michaelis–Becker Synthesis of Diethyl Phosphonates, Tetraethyl Diphosphonates, and Their Total or Partial Dealkylation. Heteroat. Chem..

[59] Souzy R., Ameduri B., Boutevin B., Virieux D. (2004). Synthesis of new aromatic perfluorovinyl ether monomers containing phosphonic acid functionality. J. Fluor. Chem..

[60] Queffeélec C., Petit M., Janvier P., Knight D.A., Bujoli B. (2012). Surface Modification Using Phosphonic Acids and Esters. Chem. Rev..

[61] Hirao T., Masunaga T., Ohshiro Y., Agawa T. (1980). Stereoselective synthesis of vinylphosphonate. Tetrahedron. Lett..

[62] Hirao T., Masunaga T., Ohshiro Y., Agawa T. (1982). Palladium-catalyzed New Carbon-Phosphorus Bond Formation. Bull. Chem. Soc. Jpn..

[63] Alexandratos S.D., Hong M.-J. (2002). Enhanced metal ion affinities by supported ligand synergistic interaction in bifunctional polymer-supported aminomethylphosphonates. Sep. Sci. Technol..

[64] Trochimczuk A.W., Jezierska J. (2000). Modification of Malonamide Ion-Exchange Chelating Resins Using the Fields Kabatschnik Reaction and Their Application to Metal Ion Removal from Aqueous Solutions. J. Inorg. Organomet. P.

[65] Papathanasiou K.E., Demadis K.D. (2015). Phosphonates in Matrices. Layered Materials Chemistry: Techniques to Tailor New Enabling Organic-Inorganic Materials.

[66] Trochimczuk A.W., Streat M. (1999). Novel chelating resins with aminothiophosphonate ligands. React. Funct. Polym..

[67] Jeanmaire T., Hervaud Y., Boutevin B. (2002). Synthesis of Dialkyl-Hydroxymethylphosphonates in Heterogeneous Conditions. Phosphorus Sulfur Silicon.

[68] Kabachnik M.I., Medved T. (1959). Vinylphosphonic acid and some of its derivatives. Russ. Chem. Bull..

[69] Macarie L., Ilia G. (2010). Poly(vinylphosphonic acid) and its derivatives. Prog. Polym. Sci..

[70] Alexandratos S.D., Strand M.A., Quillen D.R., Walder A.J. (1985). Synthesis and Characterization of Bifunctional Phosphinic Acid Resins. Macromolecules.

[71] Alexandratos S.D., Wilson D.L., Kaiser P.T. (1987). Dual mechanism bifunctional polymers: Phosphinic acid ion-exchange/redox resins and the kinetics of metal-ion complexation. React. Polym. Ion Exch. Sorbents.

[72] Trochimczuk A.W. (2001). Synthesis of functionalized phenylphosphinic acid resins through Michael reaction. Part II. Resins with multidentate ligands. React. Funct. Polym..

[73] Tavlaridesm L.L., Bae J.H., Lee C.K. (1987). Solvent Extraction, Membranes, and Ion Exchange in Hydrometallurgical Dilute Metals Separation. Sep. Sci. Technol..

[74] Beauvais R.A., Alexandratos S.D. (1998). Polymer-supported reagents for the selective complexation metal ions: An overview. React. Funct. Polym..

[75] Ferrel R.E., Olcott H.S., Fraenkel-Conrat H. (1948). Phosphorylation of Proteins with Phosphoric Acid Containing Excess Phosphorus Pentoxide. J. Am. Chem. Soc..

[76] Trochimczuk A.W. (2000). Synthesis of functionalized phenylphosphinic acid resins through Michael reaction and their ion-exchange properties. React. Funct. Polym..

[77] Bregman J.I.Y. (1952). Phosphonous and Phosphonic Cation Exchange Resins. J. Am. Chem. Soc..

[78] Boyd G.E., Vaslow F., Lindenbaum S.J. (1967). Thermodynamic Properties at 25° of Aqueous Solutions of p-EthylbenzenesuIfonic Acid and Its Alkali Metal Salts. Comparisons with Cross-Linked Polystyrenesulfonate Type Cation Exchangers. Phys. Chem..

[79] Alexandratos S.D., Quillen D.R., Bates M.E. (1987). Synthesis and Characterization of Bifunctional Ion-Exchange /Coordination Resins. Macromolecules.

[80] Sessler J.L., Melfi P.J., Pantos G.D. (2006). Uranium complexes of multidentate N-donor ligands. Coordin. Chem. Rev..

[81] Allard B., Olofsson U., Torstenfelt B. (1984). Environmental Actinide Chemistry. Inorgan. Chim. Acta.

[82] Tomiyasu H., Asano Y. (1998). Environmentally acceptable nuclear fuel cycle—development of a new reprocessing system. Prog. Nucl. Energ..

[83] Alexandratos S.D., Zhu X. (2007). High-affinity ion-complexing polymer-supported reagents: Immobilized phosphate ligands and their affinity for the uranyl ion. React. Funct. Polym..

[84] Aly M.M., Hamza M.F. (2013). A Review: Studies on Uranium Removal Using Different Techniques. Overview. J. Disper. Sci. Technol..

[85] Mincher B.J., Modolo G., Mezyk S.P. (2009). Review Article: The Effects of Radiation Chemistry on Solvent Extraction: 1. Conditions in Acidic Solution and a Review of TBP Radiolysis, Radiolysis. Solv. Extr. Ion Exc..

[86] Egawa H.T., Ikari M. (1984). Preparation of macroreticular chelating resins containing dihydroxyphosphino and/or phosphono groups and their adsorption ability for uranium. J. Appl. Polym. Sci..

[87] Egawa H., Nonaka T. (1990). Recovery of Uranium from Seawater. 7. Concentration and Separation of Uranium in Acidic Eluate. Ind. Eng. Chem. Res..

[88] Kabay N., Demircioglu M., Yaylı S., Gunay E., Yuksel M., Saglam M., Streat M. (1998). Recovery of Uranium from Phosphoric Acid Solutions Using Chelating Ion-Exchange Resins. Ind. Eng. Chem. Res..

[89] Gonzalez-Luque S., Streat M. (1983). Uranium sorption from phosphoric acid solutions using selective ion exchange resins: Part, I. Isotherms of extraction and desorption. Hydrometallurgy.

[90] Gonzalez-Luque S., Streat M. (1983). Uranium sorption from phosphoric acid solutions using selective ion exchange resins: Part II. Kinetic studies. Hydrometallurgy.

[91] Kim J., Tsouris C., Mayes R.T., Oyola Y., Saito T., Janke C.J., Dai S., Schneider E., Sachde D. (2013). Recovery of Uranium from Seawater: A Review of Current Status and Future Research Needs. Sep. Sci. Technol..

[92] Jordens A., Cheng Y.P., Waters K.E. (2013). A review of the beneficiation of rare earth element bearing minerals. Miner. Eng..

[93] Jha M.K., Kumari A., Panda R., Kumar J.R., Yo K.K., Lee J.Y. (2016). Review on hydrometallurgical recovery of rare earth metals. Hydrometallurgy.

[94] Xie F., Zhang T.A., Dreisinger D., Doyle F. (2014). A critical review on solvent extraction of rare earths from aqueous solutions. Miner. Eng..

[95] Powell J.E., Eyring L. (1964). The Separation of Rare Earths by Ion Exchange. Progress in the Science and Technology of the Rare Earths.

[96] Kabay N., Cortina J.L., Trochimczuk A., Streat M. (2010). Solvent-impregnated resins (SIRs)–Methods of preparation and their applications. React. Funct. Polym..

[97] Lederer M. (1958). Chromatography on paper impregnated with ion-exchange resins: III. The separation of some cation mixtures. J. Chromatogr. A.

[98] Juang R.S. (1999). Preparation, Properties and Sorption Behavior of Impregnated Resins Containing Acidic Organophosphorus Extractants. Proc. Nat. Sci. Counc. Rep. Chin. Part A.

[99] Alexandratos S.D., Bates M.E. (1988). Enhanced Ionic Recognition by Polymer-Supported Reagents: Synthesis and Characterization of Ion-Exchange/ Precipitation Resins. Macromolecules.

[100] Park I.-H., Rhee J.M., Jung Y.S. (1999). Synthesis and heavy metal ion adsorptivity of macroreticular chelating resins containing phosphono and carboxylic acid groups. Die Angew. Makromol. Chem..

[101] Horwitz E.P., Chiarizia R., Diamond H., Gatrone R.C., Alexandratos S.D., Trochimczuk A.W., Crick D.W. (1993). Uptake of Metal Ions by A New Chelating Ion Exchange Resin Part 1: Acid Dependencies of Actinide Ions. Solvent Extr. Ion Exc..

[102] Alexandratos S.D., Crick D.W., Quillen D.R. (1991). Development of Bifunctional Polymers for Metal Ion Separations: Ionic Recognition with Polymer-Supported Reagents. Ind. Eng. Chem. Res..

[103] Alexandratos S.D., Bates M., Walder J., McDowell W.J. (1988). Novel bifunctional resins in metal ion separations: Ion exchange/coordination resins and ion exchange/precipitation resins. Sep. Sci. Technol..

[104] Alexandratos S.D., Trochimczuk A.W., Horwitz E.P., Gatrone R.C. (1996). Synthesis and Characterization of a Bifunctional Ion Exchange Resin with Polystyrene-Immobilized Diphosphonic Acid Ligands. J. Appl. Polym. Sci..

[105] Trochimczuk A.W., Horwitz E.P., Alexandratos S.D. (1994). Complexing Properties of Diphonix, a New Chelating Resin with Diphosphonate Ligands, Toward Ga(III) and in(III). Sep. Sci. Technol..

[106] Chiarizia R., Horwitz E.P., D’Arcy K.A., Alexandratos S.D., Trochimczuk A.W. (1996). Uptake of Metal Ions by A New Chelating Ion Exchange Resin. Part 9: Silica Grafted Diphosphonic Acid. Solvent Extr. Ion Exc..

[107] Alexandratos S.D., Trochimczuk A.W., Crick D.W., Horwitz E.P., Gatrone R.C., Chiarizia R. (1996). Synthesis and Ion-Complexing Properties of a Novel Polymer-Supported Reagent with Diphosphonate Ligands. Macromolecules.

[108] Chiarizia R., Horwitz E.P., D’Arcy K.A., Alexandratos S.D., Trochimczuk A.W. (1996). Uptake of metal ions by a new chelating ion exchange resin. Part 8. Simultaneous uptake of cationic and anionic species. Solvent Extr. Ion Exc..

[109] Alexandratos S.D., Zhu X. (2015). The role of polarizability in determining metal ion affinities in polymer-supported reagents: Monoprotic phosphates and the effect of hydrogen bonding. New J. Chem..

[110] Nash K.L., Rickert P.G., Muntean J.V., Alexandratos S.D. (1994). Uptake of Metal Ions by A New Chelating Ion Exchange Resin Part 3: Protonation Constant Via Potentiometric Titration and Solid State 31P NMR Spectroscopy. Solvent Extr. Ion Exc..

[111] Chiarizia R., Horwitz E.P., Alexandratos S.D. (1994). Uptake of Metal Ions by A New Chelating Ion Exchange Resin Part 4: Kinetics. Solvent Extr. Ion Exc..

[112] Chiarizia R., Horwitz E.P., Alexandratos S.D. (1994). Uptake of Metal Ions by A New Chelating Ion Exchange Resin Part 5: The Effect of Solution Matrix on Actinides. Solvent Extr. Ion Exc..

[113] Chiarizia R., Horwitz E.P., Gatrone R.C., Alexandratos S.D., Trochimczuk A.W., Crick D.W. (1993). Uptake of Metal Ions by A New Chelating Ion Exchange Resin, Part 2: Acid Dependencies of Transition And Posttransition Metal-Ions. Solvent Extr. Ion Exc..

[114] Kilislioglu A. (2012). Ion Exchange Technologies.

[115] Yoshida M., Uezu K., Goto M., Furusaki S. (1999). Metal Ion Imprinted Microsphere Prepared by Surface Molecular Imprinting Technique Using Water-in-Oil-in-Water Emulsions. J. Appl. Polym. Sci..

[116] Yoshida M., Hatate Y., Uezu K., Goto M., Furusaki S. (2000). Metal-Imprinted Microsphere Prepared by Surface Template Polymerization and Its Application to Chromatography. J. Polym. Sci. Part A.

